# Psychosocial interventions to support parenting among parents with cancer: A scoping review

**DOI:** 10.1016/j.apjon.2026.100921

**Published:** 2026-02-19

**Authors:** Yingchun Li, Meichan Chong, Pinglei Chui, Lin Mo, Liande Tao, Yuman Yuan, Rong Liao, Haosong Ling, Qiaoli Zhong

**Affiliations:** a Department of Nursing Science, University of Malaya, Kuala Lumpur, Malaysia; b Yibin Second People's Hospital, Yibin, China; c Affiliated Children's Hospital of Chongqing Medical University, Chongqing, China

**Keywords:** Cancer, Parent, Psychosocial intervention, Social support, Communication, Scoping review

## Abstract

**Objective:**

Parents diagnosed with cancer face unique psychosocial challenges that may adversely affect parenting capacity and family functioning. This scoping review aimed to systematically map and synthesize the existing evidence on psychosocial interventions designed to support parenting and reduce psychological distress among parents with cancer.

**Methods:**

This review followed the methodological framework proposed by Arksey and O'Malley, with enhancements from Levac et al. Nine electronic databases were systematically searched from inception to June 30, 2025. Two reviewers independently conducted study selection and data extraction using predefined criteria. Eligible studies included psychosocial interventions targeting parents with cancer and reporting parenting-related or psychosocial outcomes. Data were charted and narratively synthesized.

**Results:**

A total of 53 studies met the inclusion criteria. Interventions varied considerably in theoretical foundations, delivery modes, duration, and intensity. Common components included parent–child communication support, psychoeducation, social support facilitation, coping skills training, and self-efficacy enhancement. Most interventions demonstrated positive trends in improving parenting confidence, communication quality, emotional adjustment, and family functioning. However, few interventions were culturally tailored or specifically designed to address developmental stages of children or diverse family structures. Digital and web-based approaches were emerging but remained limited.

**Conclusions:**

Psychosocial interventions show promise in supporting parenting and psychosocial well-being among parents with cancer. Nevertheless, significant gaps remain in cultural adaptation, personalization, and scalability. Future research should prioritize theory-informed intervention development, inclusion of underrepresented populations, and the integration of digital health strategies to enhance accessibility and sustainability.

## Introduction

With recent medical progress, cancers are being diagnosed at increasingly younger ages. A recent review shows that 14%–24.7% of patients with cancer are parents of underaged children or young adults, and that 1.6% to 8.4% of children, adolescents, and young adults have parents with histories of cancer.^1^ Patients with cancer face significant challenges in balancing the demands of managing their illness with the responsibilities of parenting underage children.^2^ This dual burden often results in considerable psychological distress, encompassing concerns about the psychological impact of their illness on their children, the parenting competence of other caregivers, and fears regarding the transmission of cancer to their children.^3^ These concerns, collectively referred to as “parenting concerns” (PC) or “child-rearing concerns,” highlight the emotional strain experienced by cancer patients as they navigate the complexities of parenting while undergoing diagnosis and treatment.^4^ Research indicates that PC is prevalent among patients with cancer and their spouses, persisting throughout the course of diagnosis and treatment and significantly influencing their mental and physical health as well as medical decision-making.^5^ Studies report that 24%–71% of patients with cancer experience anxiety and depression related to parenting concerns, which can exacerbate their psychological distress, impede participation in medical care, and negatively impact treatment outcomes, leading to longer hospital stays;^6^^,^^7^ These findings underscore how PC can become a major obstacle to the rehabilitation and quality of life of cancer patients.

Confronted with this practical challenge, implementing targeted psychosocial interventions is of significant importance. Psychosocial emphasizes the integration of individual psychological development and social interactions, with its interventions encompassing both psychological and social dimensions of treatment.^8^ Psychology, as the scientific discipline that studies psychological phenomena and their underlying principles, provides theoretical support for related interventions. Among these, psychosocial interventions specifically refer to non-pharmacological approaches—such as psychotherapy, cognitive behavioral therapy, and mindfulness training—that aim to improve patients' emotional states, cognitive patterns, and behaviors.^9^ Evidence suggests that psychosocial interventions can have a positive impact on the psychological and physical well-being of patients with cancer raising underage children, as well as on their children's outcomes.^2^ These interventions aim to address psychological and emotional well-being by enhancing social functioning, interpersonal relationships, and spiritual dimensions,^10^ which can support parents in issues regarding open communication about cancer within the family or age-appropriate information about cancer. Moreover, parents can be supported emotionally and can be reassured in their parenting competence.^11^ Yet, studies shown that parents often seek not only informational guidance related to cancer care but also a greater level of emotional understanding and coping strategies. In contrast, healthcare providers might predominantly focus on conveying medical information and offering general psychosocial resources, which might not fully cater to the emotional challenges parents face in balancing cancer diagnosis and parenting responsibilities.^12^ Given the growing number of people reporting distress associated with the impact of cancer on their parenting and co-parenting roles, the provision of psychological interventions to both parents in the cancer context has become a major issue. In recent years, interventions aiming to facilitate parents' support of their children have been proposed. However, evidence in summarizing the interventions are still in their nascent stage.

Existing psychosocial interventions developed for parents diagnosed with cancer and their families have shown feasibility and preliminary effectiveness in improving parental quality of life.^13^, ^14^, ^15^ However, as the limited scope of methodologically rigorous studies currently available precludes definitive conclusions about intervention efficacy.^16^ Existing reviews have established the effectiveness of psychosocial interventions in supporting children affected by parental cancer,^17^ and have explored whether such interventions align with the developmental needs of these children.^18^ However, they have not sufficiently addressed the impact of interventions on the parents themselves, and few studies have simultaneously evaluated the comprehensive effects of interventions across multiple dimensions such as parents, and families. Furthermore, while previous reviews in the context of parental cancer have predominantly concentrated on those with advanced illness,^19^^,^^20^ they have often overlooked parents in earlier phases of the disease. Although the recent reviews highlighted the diversity of available psychosocial interventions for parents with cancer and the outcomes on parenting distress, as well as methodological challenges,^21^, ^22^, ^23^ the literature search for both reviews ended in 2023. More interventions have been developed since the previous reviews.^15^^,^^24^^,^^25^ Additionally, the development process of complex interventions often takes time.^26^ Previously developed interventions have been evaluated after the previous reviews.^27^^,^^28^ Hence, an updated synthesis of current psychosocial interventions designed for parents with cancer is needed to comprehensively map the existing landscape and inform the future development, assessment, and implementation of supportive programs for affected parents and their families.

Given the complexity of this issue, this scoping review seeks to map and synthesize emerging evidence in the field. It aims to identify common themes in the psychosocial interventions of patients with cancer who are parents, while highlighting gaps in the literature to inform the development of future interventions.

## Methods

### Review design

This scoping review is based on the five-stage framework proposed by Arksey and O'Malley^29^ and systematically incorporates six key enhancements suggested by Levac et al.^30^ to this framework, which include: (1) strengthening research positioning by clearly linking research objectives with specific questions; (2) optimizing the search process through iterative search techniques (MIST) and snowballing to enhance transparency and comprehensiveness in literature coverage; (3) introducing team collaboration and iterative mechanisms to complete literature screening and data extraction through multiple rounds of discussion; (4) deepening result analysis by integrating quantitative statistics with qualitative thematic analysis and highlighting implications for policy, practice, and research; (5) expanding knowledge translation by incorporating stakeholder consultations into the research process; and (6) standardizing data extraction to systematically address study design, scope of application, and methodological limitations. The adoption of these enhancements aims to improve the rigor of literature screening, enhance the interpretability of heterogeneous evidence, and ensure the practical translational value of the review findings. The scoping review protocol was registered on the Open Science Framework (OSF, Registration No. https://doi.org/10.17605/OSF.IO/5B9TU) and adhered to the Preferred Reporting Items for Systematic reviews and Meta-Analyses extension for Scoping Reviews (PRISMA-ScR) guidelines.

### Identification of the research questions and objectives

The primary research question guiding this scoping review was: What psychosocial interventions are used to improve parenting-related psychological issues in patients with cancer, and what are their benefits? The objectives of the review were to provide a comprehensive overview of psychosocial interventions designed to address these parenting-related psychological issues in patients with cancer and to identify the contents and outcomes of these interventions.

### Search strategy

The search strategy was developed based on the PICO framework (Population, Interest, Context), as outlined by Stern et al.,^31^ to ensure a comprehensive exploration of relevant studies. A systematic search was conducted across English databases including PubMed, Embase, the Cochrane Central Register of Controlled Trials (CENTRAL), Cumulative Index to Nursing and Allied Health Literature Complete (CINAHL), PsychINFO, Social Work Abstracts, Web of Science, as well as Chinese databases such as China National Knowledge Infrastructure (CNKI), and Wan Fang Data. These searches covered all publications up to June 30, 2025.

Key terms for searching included: (neoplasm∗ or cancer∗ or tumor∗ or oncol∗ or onto∗ or carcino∗); (mother∗ or father∗ or partner or parent∗ or paternal or maternal or father-child or mother-child or parents or famil∗); (psychological∗ or psychosocial∗ or social∗ or behavior∗ or cogniti∗ or parenting∗ or emotion∗ or psychoeducation∗ or education∗ or coping∗ or adjustment∗); (intervention∗ or support∗ or program∗ or group∗ or therap∗ or counsel∗). Both free-text terms and Medical Subject Headings (MeSH) were employed where applicable. A snowball search was also performed to identify additional relevant articles by reviewing the reference lists of included studies. Details of the full search strategy are provided in **Supplementary File 1.**

### Eligibility criteria

Publications with any study design were included if they described (a) psychosocial interventions that (b) targeted patients with cancer parenting at least one child under the age of 18, and (c) targeted improving psychological factors such as parenting concerns, depression, anxiety, emotional functioning, and psychological distress, as well as social factors such as parenting self-efficacy, parenting skills, parental capacity, quality of life, and family functioning. (d) studies published in English or Chinese in peer-reviewed journals were included. Exclusion criteria were Studies were excluded (a) if they focused solely on pharmacological interventions, medical directives, training or education of health professionals, or assessments of psychosocial needs without intervention. (b) studies addressing only biological, physiological, or survival outcomes. (c) psychosocial interventions involving only children, and (d) no explicit connection to parental mental health.

### Data extraction and analysis

All identified articles from the searches were exported into EndNote × 21.0 for duplicate removal and initial screening of titles and abstracts. Two reviewers (LYC and LHS) independently assessed the studies for inclusion based on the eligibility criteria. Any disparities in data extraction were resolved through discussion with a third reviewer (ML). A data extraction framework, developed in line with the scoping review questions and JBI guidelines,^32^ was used to organize the data. Extracted information included authorship details, year of publication, country, participant characteristics, sample size, intervention title, intervention aim, theoretical framework, features of psychosocial interventions, attrition rate, outcome measures, and study results. Pilot testing of the framework was conducted before full application to ensure its reliability. The extracted data were then synthesized, summarized textually, and organized into key categories, which were presented in tabulated form.

### Reporting bias assessment

To assess risk of bias in the included studies, the revised Mixed Methods Appraisal Tool (MMAT) was used.^33^^,^^34^ Risk of bias was assessed with five relevant criteria for different study designs, where each criterion was converted into a percentage for comparison (e.g., if the study fulfilled one criterion out of five, the quality of the study was 20%, i.e., a high risk of bias). The higher the percentage, the lower the risk of bias was present. Prior to formal assessment, three reviewers (CMC, LYC, LHS) pilot-tested the tool to ensure consistency in its application. Subsequently, two reviewers (LYC, LHS) independently evaluated the full studies, and discussed any ambiguities during the assessment.

## Results

### Study selection

**Fig. 1:**
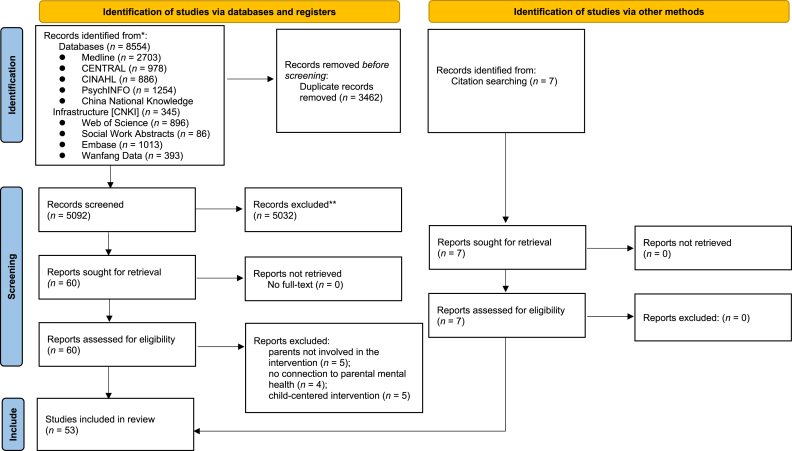
Flow diagram illustrating the original process of screening and identification of studies.

Electronic searches of databases identiffed 8554 records. The duplicates were removed (*n* = 3462) and the remaining records were screened (*n* = 5092). After screening for titles and abstracts, a total of 5032 articles were excluded due to reasons such as mismatches in study type (e.g., reviews, conference papers), study population (non-cancer patients), or intervention type (non-psychosocial interventions). Subsequently, 60 articles were further screened for full text in which 14 were excluded mainly because of parents not involved in the intervention (*n* = 5), no connection to parental mental health (*n* = 4) and child-centered intervention (*n* = 5). Seven additional records were identified by citation searching. Finally, 53 studies were included in this scoping review. Fig. 1 shows the PRISMA-ScR flow chart of study selection.

### Study characteristics

Table 1 summarizes the characteristics of the 53 studies, which were published between 2003 and 2025, and were conducted in the United States (*n* = 20), Germany (*n* = 6), Australia (*n* = 5), Sweden (*n* = 4), Norway (*n* = 3), Japan (*n* = 3), Switzerland (*n* = 3), Denmark (*n* = 2), Finland (*n* = 2), Israel (*n* = 1), China (*n* = 2) , Iran (*n* = 1), and France (*n* = 1). The studies encompassed a range of designs, including 9 randomized controlled trials (RCTs), 25 pre-post intervention studies, 13 qualitative studies, 5 mixed-methods studies, and one case report.

**Table 1:** Summary of intervention characteristics and main findings for parental outcomes.

Author (Year) Country	Study design	Participants	Intervention title	Intervention aims	Theoretical Framework	Intervention			Attrition rate	Outcome measurement and assessment tools	Main findings	Quality criteria met
						Components	1. Average time per Session 2. Frequency 3. Duration	1. Delivery Mode 2. Intervention modalities 3. Structure 4. Intervention facilitators 5. Setting				
Davis Kirsch et al. (2003) USA	Qualitative evaluation study	4 mother-child dyads	Making the most of the moment (pilot study for the enhancing connections program)	Enhance mother-child relationship quality during breast cancer treatment. Improve communication about cancer within families. Reduce children's fears/anxiety related to maternal illness. Support mothers in balancing parenting and self-care.	A developmental–contextual model of parenting, coping theory, and social cognitive theory	**Psycho-educative elements and homework assignments in 5 sessions:** **Mother-focused sessions:** Education on cancer communication, child development, and coping strategies. **Mother-child activities:** Workbook assignments (e.g., “stop and think” exercises). Role-playing to practice open-ended questions. Legacy-building activities (e.g., creating memory books).	1. Not specified 2. Weekly 3. 5 weeks	1. Face-to-face with the family 2. Workbooks 3. Combined individual mother coaching + joint mother-child activities 4. Nurse 5. Home	0	Structured interview	Mothers described skills they learned, such as use of open-ended questions, as particularly helpful in enhancing the mother–child relationship. Fathers claimed that at-home assignments enriched the mother-child relationship and that the mother spent more time with the child. The educational effort of the programme was empowering for the mothers.	100%
Brandt et al. (2004) USA	Implementation (pilot study) pre-/post-test design	8 mothers, 5 fathers, 8 children	No information	Improve the quality of mother-child relationship, improve parenting behaviour	Social cognitive theory	**Psycho-educative and coping skill in 5 sessions:** The content of the intervention included an emphasis on the mothers' self-care and their listening skills with a focus on the children's concerns and coping efforts related to the breast cancer experience.	1. No information 2. No information 3. 60 minutes	1. No information 2. No information 3. Parent-centred 4. Clinician 5. Home	0	Structured interview	Improve the quality of mother-child relationship, improve parenting behaviour	40%
Christ et al. (2005) USA	RCT	184 child-parent dyads, Intervention: 79, Control: 47	“Parent guidance intervention"	(1) Sustain competence beyond the time of the intervention in providing support and care for the children. (2) Provide an environment in which the children felt able to express painful or conflicting feelings, thoughts, and fantasies about the loss. (3) Maintain consistency and stability in the children's environment.	Parent guidance model	**Emotional expression, communication, and coping strategies 6 sessions:** (1) Emphasize background data about each parent and child and about the cancer illness; (2) Emphasize the therapeutic engagement; (3)–(6) interventionist engaged in problem solving around the immediate crisis of the patient's deteriorating condition; talk about ways of handling emerging problems with the children and current approaches to communication about the terminal illness.	1. 60–90 min 2. No information 3. 12 months	1. Face to face 2. No information 3. Therapeutic interviews in the home environment 4. Clinical team: one social worker along with their supervisors 5. Home	10.18%	**Parenting coping:** Stress associated with events, ability to express feelings and mental health **Child-report parental competence/communication:** POPM	Parenting coping ↑ Child-report parental competence/communication ↑ (*P* < 0.01)	100%
Thastum et al. (2006) Denmark	Pre-/post intervention study	40 families, Intervention: *n* = 24, Control: *n* = 16	The counselling project (COSIP study)	Enhance parenting competence, support the parents in age appropriate communication and support the parents' use of possible network.	Stress and coping theory	**Psycho-educative and** counselling **in 5**–**6 sessions:** (1) discuss the counselling goals and framework with the parents; (2)–(6) counselling focused on illness-related problems and well-defined goals.	1. No information 2. No information 3. 4.7 months	1. Face-to-face with the family 2. No information 3. Focused, short term and needs based sessions with the family. 4. One counsellor trained in systemic family therapy and one psychotherapist. 5. Variable	29.41%	**Parent's depression:** BDI-II: 21 **Family functioning:** FAD: 53	Parent's depression ↓ (*t* = 2.58, *P* < 0.05) FAD communication ↑ (*t* = 3.18, *P* < 0.005)	60%
Lewis et al. (2006) USA	Pre-/post intervention study	13 breast cancer patients and their 13 school age children	The enhancing connections programme (EC)	Respond to the documented experiences and sources of distress in both mothers and children impacted by maternal breast cancer.	A developmental-contextual model of parenting, the transtheoretical model of coping, and Bandura's social cognitive theory	**Psycho-educative, reflective and skill- and efficacy-building elements in 5 sessions:** (1) Five scripted patient education sessions delivered at 2-week intervals to the mother; (2) An interactive booklet about breast cancer to be read by the mother to the child; (3) A mother's workbook containing didactic text as well as in-session and at-home scripted assignments between the mother and child; (4) The child's ‘my story’ booklet for drawing and adding information about the child's interests and ways of dealing with stresses; (5) Access by phone pager to a patient educator for 12h each day for contact as needed between the home-based interventions.	1. No information 2. Every 2 weeks 3. 10 weeks	1. Face-to-face with mother and child 2. Workbook; booklet 3. Child-, parent-, family-parenting education 4. Patient educator 5. Home	0	**Mother's parenting quality:** FPRQ **Mother's mood and anxiety:** CES-D **Mother's self-efficacy:** CASE	Pre–post-test differences showed improvements in the mother's depressed mood; state anxiety; and self-efffcacy. There was no signiffcant improvement in the quality of the mother–child relationship. Mothers claimed they gained ways to: (1) manage their emotions in the moment when interacting with their child; (2) add to their self-care; (3) listen to their child better; and (4) better understand their child's behaviour.	60%
Kissane et al. (2006) Australia	RCT	81 families of cancer patients, Intervention: 53, Control: 28	Family focused Grief therapy	Optimize cohesion, communication, and handling of conflict, promote the sharing of grief and mutual support	No information	**Psycho-educative, skill- and efficacy-building elements in 4**–**8 sessions:** (1) Assessment (one or two weekly sessions) concentrates on identifying issues and concerns relevant to the specific family and on devising a plan to deal with them. (2) Intervention (typically two to four sessions) focuses on the agreed concerns. (3) Termination (one or two sessions) consolidates gains and confronts the end of therapy.	1. No information 2. Variable 3. 90 minutes	1. No information 2. A manual for conducting the therapy and then published this in a book as a series of guidelines 3. Family-based 4. Trained health professionals specializing in cancer care: 4 nurses, 1 sociologists, and 1 art therapists. 5. Hospital or home	15.09%	**Family functioning:** FAD **Psychological morbidity:** BSI **Cognitive:** BDI	The overall impact of family focused grief therapy was modest, with a reduction in distress at 13 months. Significant improvements in distress and depression occurred among individuals with high baseline scores on the brief symptom inventory and beck depression inventory. Global family functioning did not change. Sullen families and those with intermediate functioning tended to improve overall, whereas depression was unchanged in hostile families.	100%
Schmitt et al. (2007) Finland	Pre-/post intervention study	37 cancer patients and their families	Preventive Counselling Service (COSIP), Finland	Support parenting and parenthood, assess need of all family members, accompany family members in process through loss and grief.	No information	**Emotional expression, communication, and coping strategies in 5**–**6 sessions:** 1-2 family sessions. 1-2 couple sessions. 1 sibling session. 1 individual session per child. Optional follow-up sessions.	1. 50–60 minutes 2. Varied based on family needs 3. Varied	1. Face to face 2. Brochures, phone interviews 3. In-person sessions 4. Two experienced family therapists per session, with specialties in child psychiatry and cancer care. 5 hospital	69%	A one-page questionnaire	Families valued the intervention, suggesting it should be routine for cancer patients with children.	60%
Bugge et al. (2008) Norway	Qualitative design	13 parents (6 fathers and 7 mothers), 12 children were aged between 6 and 16 years, mean age of 9 years, 8 girls and 4 boys	“Family talks in cancer care” program (family support program	Prevent psychosocial problems, promote coping, help to talk about disease, knowledge and information about disease, help to plan for the future	Family resilience theory, coping theory for children	**Psycho-educative, reflective and skill- and efficacy-building elements in 5 meetings:** (1) A short meeting involving presentation and introduction (2) Parents' meeting: Talking with parents about their experiences with parenting and family coping during the illness situation (3) Children’ s meetings: Talking with each child in the family about his/her experiences and changes during the illness situation. (4) Family meeting: Children and parents discussed concerns and assessed family strengths. (5) Family meeting: Discuss how to face the future using the family's strengths and where to seek additional help or support if needed.	1. No information 2. No information 3. 6 weeks	1. Face to face 2. Books and films 3. Family individualised intervention. 4. A team of six health workers specialising in cancer care (four nurses, a sociologist and an art therapist) with two workers based in each hospital 5. Hospital	40%	In-depth interviews/after intervention	(1) Support the family pulling together by increasing open communication, parents' understanding and support strategies for their children; (2) reframing the crisis by identifying and using family strengths in the illness situation; (3) help in planning the future and the help needed	80%
Bugge et al. (2009) Norway	Qualitative design	6 families (12 children) parents age 34–54, 6 patients, with all together 12 children age 6–16, the location of cancer included: Breast/brain/bowel/pancreas	“Family talks in cancer care” program (family support program	Prevent psychosocial problems, promote coping, help to talk about disease, knowledge and information about disease, help to plan for the future	Family resilience theory, coping theory for children	**Psycho-educative, reflective and skill- and efficacy-building elements in 5 meetings:** (1) A short meeting involving presentation and introduction (2) Parents' meeting: Talking with parents about their experiences with parenting and family coping during the illness situation (3) Children’ s meetings: Talking with each child in the family about his/her experiences and changes during the illness situation. (4) Family meeting: Children and parents discussed concerns and assessed family strengths. (5) Family meeting: Discuss how to face the future using the family's strengths and where to seek additional help or support if needed.	1. No information 2. No information 3. 6 weeks	1. Face to face 2. Books and films 3. Family individualised intervention. 4. A team of six health workers specialising in cancer care (four nurses, a sociologist and an art therapist) with two workers based in each hospital 5. Hospital	40%	Interviews/took place up to six weeks after the programme was finished	(1) Confidence to talk about the illness situation, (2) talking about family strengths, confirmation, and association within the family, (3) information sharing with social network about the illness situation,(4) increased knowledge about the illness and prognosis, (5) being important and valuable in the family, (6) confronting and coping with their own fear and other reactions to illness situation	80%
Werner-Lin and Biank (2009) USA	Qualitative study	Parents and their children (5–11 years); number not specified	“Family matters"	Normalise emotions/experiences; reshape the family system to maximize resources and build on areas of vulnerability	Family systems theory, developmental theory, attachment theory	**Psycho-educative and activity elements in 2 modules:** (1) Support parents (2) Children share their work with parents	1. 1 hour (separate) + 15-min joint sharing 2. Weekly or biweekly meetings 3. Varied by group	1. No information 2. No information 3. Family-centered group; couples support group 4. A team of health care professionals: social workers, psychologists, dieticians, nurses, exercise specialists, and marriage and family therapists 5. No information	20%	Parents communicate needs and feelings to loved ones: Open-ended interviews	Parent/child self-reports of improved communication, reduced anxiety	40%
Hasson-Ohayon and Braun (2011) Israel	Qualitative study	12 cancer patients or partners	Being a parent and coping with cancer (PCWC)	Empower the patients and spouses in their parenting role, and to help the parents help their children to adjust and cope	No information	**Psycho-educative and activity elements in 4 sessions:** (1) Telling and sharing (2) Children’ responses (3) Routine and changes (4) Learning and Awareness as a parent.	1. One day workshop 2. No information 3. One day	1. Face-to-face in groups 2. workbook 3. Parents group workshops 4. No information 5. Clinic	0	Questionnaires and semi-structured interviews	Parents reported it to be helpful in empowering them as parents and in imparting learning tools for identifying their children's needs, as well as for communicating with their children.	40%
Niemelä et al. (2012) Finland	Pre-/post intervention study	19 families (19 parents with various cancer types, 15 spouses and 32 children aged 8–17)	Struggle for life trial	No information	No information	**Psycho-educative, reflective and counselling elements in 2 modules:** Let's talk about the children intervention (LT): Psycho-education and counselling in 2 sessions. The family talk intervention (FTI): Psycho-educative, reflective and counselling elements in 6–8 sessions	1. No information 2. No information 3. No information	1. No information 2. No information 3. LT: Parents only; FTI: Family members 4. clinicians 5. No information	47%	**Psychiatric symptom:** SCL-90 questionnaire	A decrease was found in overall psychiatric symptoms at 4- months follow-up among patients, and specifically for symptoms of anxiety and hostility. (Mean change, −0.35, *t* = 2.64, *P* = 0.039) and spouses (−0.19, *t* = 2.73, *P* = 0.029)	40%
Davey et al. (2013) USA	Pre-/post intervention study	12 parents with various cancer types and 19 children aged 10–18, Intervention group: 7 families; Control group: 5 families	A culturally adapted interactive family-focused programme	Give culturally sensitive psychosocial support to African-American families with parental cancer	Attachment theory. Clarke's school-age child support group model and Beardslee's preventive intervention model	**Psycho-educative, emotional expression, reflective and skill- elements in 5 sessions:** (1) Assist mothers in managing cancer-related emotions and enhancing self-care skills; (2) Increase listening skills; (3) Application of listening skills; (4) Evaluate a child's coping strategies and measures that can be taken to cope with cancer-related stress; (5) Self-reflection exercises help mothers build confidence.	1. From 90 min to 2 h 2. Bi-monthly 3. 10 weeks	1. Face-to face in groups 2. No information 3. Dyads, family group support. Manualised 4. An African American female therapist 5. Home	0%	**General communication:** 10 questions, based on the work of barnes and Olsen **Parent–adolescent relationship:** IBQ **Parent's depression:** CES-D	Significant improvement in parent-child communication (*Z* = −1.89, *P* = 0.056). No significant changes in parent–adolescent relationship and parent's depression.	60%
John et al. (2013) Germany	Pre-/post intervention study	116 breast cancer mothers; 116 dependent children (3–14 years)	‘Getting well together’	Prevent at-risk children from developing serious emotional and behavioural problems	Resource-oriented positive psychology, stress and coping research, systemic solution focused therapy, and the COSIP (children of somatically Ill parents) manual	**Psycho-**educative**, emotion regulation,** skill**- and supportive elements 11 sessions: (1) 2 sessions for mother:** Promote open and age appropriate family dialogue, provide support, convey the principles of communication and precautions for communication through psychological education and face-to-face and resource group dialogue. **(2) 1 sessions for fathers/partners:** Provide ways to ease emotions, provide specific coping methods related to breast cancer, marriage support and family communication. **(3) 4 sessions ((including one mother-child session) for preschoolers:** Promote children's relaxation, enhance mother child interaction, carry out mother child massage, guided visualization, and creative handicraft activities. **(4) 4 sessions for school aged children:** Develop coping strategies, provide a safe environment for conveying fears or questions about their mother's illness, and enhance self-confidence through storytelling, model-based learning, discussions, games, and other means;	1. Mothers: 90 minutes; Fathers/Partners: 2 h; preschoolers: 45 minutes; school children:90–180 minutes; 2. No information 3. 3 weeks	1. Face-to-face 2. No information 3. Family-oriented group intervention 4. No information 5. Hospital	50%	**Mother's** quality **of life:** EORTC QLQ-C30	Differences in parental outcome achieved during the programme (intervention period, Pre2–Post) exceeded the differences achieved prior to participation (waiting period, Pre1–Pre2) for mothers' emotional functioning. Mother's health-related quality of life improved more during the intervention than during the time period before the intervention.	60%
Lewis et al. (2015) USA	RCT	176 mothers with breast cancer and 176 children aged 8–12, Intervention group: 90 mother-child dyad, Control group: 86 mother-child dyad	The enhancing connections Programme (EC)	Decrease maternal depressed mood and anxiety, improve parenting behaviour (parenting quality, skills and self-efficacy), and improve children's behavioural emotional adjustment to their mother's breast cancer	A developmental contextual model of parenting, the transtheoretical model of coping, and Bandura's social cognitive theory	**Psycho-educative, reflective and skill- and efficacy-building elements in 5** sessions**:** (1) Assist mothers in managing cancer-related emotions and enhancing self-care skills; (2) Increase listening skills; (3) Application of listening skills; (4) Evaluate a child's coping strategies and measures that can be taken to cope with cancer-related stress; (5) Self-reflection exercises help mothers build confidence.	1. 10 or fewer minutes 2. 2-week intervals 3. No information	1. Intervention group: Face-toface with mother and child. Control group: Telephone with mother 2 .interactive booklet about breast cancer; a mother's workbook; a child-completed activity booklet 3. Home-based intervention 4. Patient educator 5. Home	43%	**Depression:** CES–D **Anxiety:** STAI **Mother's self-efficacy:** CASE **Parenting quality:** FPRQ **Parenting skills:** The parenting skills checklist developed for the study	All significant changes occurred at 2 months, but improvements were not significant at 12 months. Compared with the control group, mothers in the intervention group had fewer depressive symptoms (Cohen's d = 0.29), improved parenting skills (Cohen's d = 0.32), and lower anxiety (Cohen's d = 0.26). Mothers in the intervention group tended to have greater confidence than controls on the help child subscale (Cohen's d = 0.25) and tended to score higher on parenting quality on Disclosure of negative feelings (Cohen's d = 0.30).	20%
Landry-Dattée et al. (2016) France	Qualitative study	61 families (71 adults, 19 children)	Child–parent support group	(1) Facilitate communication (2) Help support child and their symptoms	Psychoanalytic theory; clinical practice	**Psycho-educative and discussion elements in 2** sessions**:** (1) A 15-min film which addressing the topics of cancer, the main treatments, the possibility of death, sadness, guilt, impotence, and the fear of separation. (2) 1-h discussion.	1. 2 hours 2. 2-week interval 3. No information	1. Face-to-face 2. Movie; book 3. Mother-child group 4. psychologist 5. Hospital	0	Post-intervention semi-directed qualitative interview	Helped facilitate communication about cancer with children; peer support; parents perceived reduced distress symptoms in children	40%
Kobayashi et al. (2017) Japan	Pre-/post intervention study	24 parents (23 mothers and 1 father) diagnosed with various cancer types and 38 children aged 6–12	CLIMB programme	Reduce parents' anxiety and distress related to their child's stress, and improve communications between parents and children.	Principles of mental health promotion	**Psycho-educative and discussion elements and assignments in 6** sessions**:** (1) “All about me” -introduce themselves (2) “What is cancer?” -Learn about cancer and its treatment (3) “Feeling mask”-Make a mask to express sad feelings (4) “Strong box”-Make a box to feel strength (5) “Anger cube”-Make a cube to deal with anger (6) “Get well card”-Write a get well note to parent with cancer	1. 2 hours 2. Weekly 3. 6 weeks	1. Face-to-face 2. No information 3. Parents-children group 4. Psychosocial oncology professionals: social worker, psychologist and child-life specialist 5. Hospital	25%	**Quality of life:** FACIT–Sp **Anxiety and depression:** HADS **Posttraumatic stress symptoms:** IES-R	Parental quality of life improved after the group intervention with respect to social/ family well-being; emotional well-being; functional well-being; and spiritual well-being. No signiffcant changes were identiffed on physical well-being, nor pre- and posttest anxiety and depression scores (total: *t* = 20.40, *P* = 0.690). No signiffcant changes were found in posttraumatic stress symptoms.	60%
Lewis et al. (2017) USA	Pre-/post intervention study	32 mothers with breast cancer	The enhancing connections telephone (EC-T) programme	Decrease maternal depressed mood and anxiety, improve parenting behaviour (parenting quality, skills and selfefficacy), and improve children's behavioural emotional adjustment to their mother's breast cancer.	A developmental-contextual model of parenting, the transtheoretical model of coping, and Bandura's social cognitive theory	**5** sessions: (1) assist mothers in managing cancer-related emotions and enhancing self-care skills; (2) Increase listening skills; (3) Application of listening skills; (4) Evaluate a child's coping strategies and measures that can be taken to cope with cancer-related stress; (5) Self-reflection exercises help mothers build confidence.	1. 30–60 minutes 2. 2-week intervals 3. 10 weeks	1. Telephone 2. No information 3. Mother only 4. Patient educator 5. Home (via telephone)	27.27%	**Depressed mood:** CES-D **Anxiety:** STAI **Parenting self-efficacy:** CASE **Parenting quality:** FPRQ **Parenting skills:** The parenting skills checklist developed for the study	Maternal depressed mood did not significantly change but showed a tendency for improvement. However, maternal anxiety improved between baseline and post-intervention. Parenting competencies improved on both parenting skills and parenting self-efficacy. Parenting skills improved significantly as well as mothers' selfefficacy. Parenting quality did not significantly change but remained stable between the pre- and post-test scores. Mothers said their greatest gains were in acquiring and practising new ways to communicate with their child.	80%
Fife et al. (2017) USA	Pre-/post intervention study	60 families with a parent with cancer undergoing bone marrow transplant (BMT) and children aged 10–18, Intervention: 31 families; Control: 29 families	Brief family intervention	Reduce emotional distress, facilitate supportive functioning within the family and promote open communication	No information	**Psychoeducation and counselling in 3** sessions**:** (1) Dyadic sessions for BMT recipients and caregiver partners. (2) Individual session for caregivers. (3) Digital video discs (DVDs) for children (ages 10–18).	1. Dyadic sessions: 40–45 minutes per session; child interviews: 40 minutes. 2. Dyad sessions: 2 sessions (pre-hospitalization and post-discharge); caregiver session: 1 individual session during hospitalization; child DVDs: 2 DVDs (pre-hospitalization and post-discharge) 3. The intervention spanned from pre-hospitalization to 4 months post-discharge.	1. Face-to-face and via telephone individually 2. Manualized intervention for consistency; DVDs for children. 3. Dyadic and single 4. Health professionals 5. NCI-designated cancer center and community hospital.	51.60%	**Emotional distress:** PANAS **Avoidance coping:** Ways of coping Checklist Avoidance subscale; RSQ **Family communication:** FES **Dyadic adjustment:** DAS	At 4 months, within-group analyses, the intervention group experienced an increased sense of family cohesion, decreased emotional distress at 1 month (Cohen's d = 0.30) and 4 months (Cohen's d = 0.47), and for the control group at 4 months (d = 0.27). There was less avoidance coping in the intervention group at 1 month (Cohen's d = 0.33). Summarising between-group results at 1 and 4 months, small effect sizes were seen favouring the intervention group for family cohesion at 4 months (d = 0.38), emotional distress at 1 month (d = 0.21) and 4 months (d = 0.22) and avoidance coping at 1 month (d = 0.40).	60%
Stafford et al. (2017) Australia	Study protocol pre-/ post intervention study	Planned: Parents with cancer who have children aged 3–12	Enhancing Parenting in cancer (EPIC)	Improve parenting efficacy and reduce parental stress and enhance family communication	Attachment theory and social cognitive theory	**Psycho-educative and reflective** elements **in 3 modules:** (1) Psycho-educational DVD: Patient interviews and expert commentary. (2) Question prompt list (QPL): Tailored questions for healthcare providers. (3) Telephone call: Follow-up with a clinical psychologist for consolidation and referrals.	1. DVD: 30–60 min (self-paced); QPL: Self-administered; phone call: 30 min. 2. Single intervention delivery (DVD + QPL mailed; phone call 2 weeks later) 3. Pre-intervention to 1-month post-intervention follow-up.	1. DVD (mailed/online), QPL (paper/digital), phone call (scheduled) 2. DVD (pre-recorded), QPL (structured list) 3. Single 4. Clinical psychologist (phone call); DVD/QPL self-administered 5. Home	NA	**Parenting concerns:** PCQ **Parental** stress**:** PSI-R SF **Parenting self-efficacy:** PSOCS **Family functioning:** FAD **Parental psychological Morbidity:** DASS-21	None, study protocol.	NA
Walker et al. (2018) USA	Qualitative evaluation study	31 mothers with various types of cancer and 31 children aged 5–12	Enhancing Connections Telephone Programme (EC-T)	Enhance mothers' ability to be emotionally present for their children. Improve maternal skills in communicating with children about cancer.	A developmental-contextual model of parenting, the transtheoretical model of coping, and Bandura's social cognitive theory	**Psycho-educative, reflective and skill- and efficacy-building elements in 5** sessions (1) Assist mothers in managing cancer-related emotions and enhancing self-care skills; (2) Increase listening skills; (3) Application of listening skills; (4) Evaluate a child's coping strategies and measures that can be taken to cope with cancer-related stress; (5) Self-reflection exercises help mothers build confidence.	1. 30–60 minutes 2. 2-week intervals 3. 10 weeks total (from first session to post-intervention follow-up	1. Telephone 2. Manualized scripts 3. Patient single 4. Oncology nurses trained in the EC-T protocol. 5. Remote (home-based via telephone)	0	Semi-structured interview	Mothers described that they understood the children's perspective, learnt how to handle their children in a better way, their communication with the children about cancer improved and they were more Emotionally available to their children.	40%
Senneseth et al. (2018) Norway	RCT	35 cancer patients or partners; Children, Intervention: 17, Control: 18	“Cancer-PEPSONE programme (CPP)"	Improve psychological distress, quality of life and parental capacity.	Social network support model	**Psycho-educative and discussion elements and assignments in 2** sessions**:** (1) Psychoeducational session: Providing information on the challenges faced by families dealing with parental cancer; covers reactions and needs of parents and children, importance of social support, and how to provide effective support. (2) Discussion session: The families are encouraged to express their specific support needs, social network members (supporters) are encouraged to state the types and frequencies of support to which they can commit.	1. 180 minutes 2. Single-session 3. 180 minutes	1. Face to face 2. NR 3. A single-session social network meeting for each family individually. 4. Psychologist 5. Home or the other place	40%	**Received** social **support:** CSS **Perceived social support:** MSPSS **Psychological distress:** GHQ-12 **Quality of life:** QOLS-N **Parental capacity:** SEPTI	CPP may help parents in maintaining social support and enhancing parental capacity.	100%
Bingisser et al. (2018) Switzerland	Case report	1 family (mother with breast cancer, father, 2 sons aged 8 and 11)	FAMOCA (family online counselling for families with parental cancer)	Improve psychological adjustment in families, enhance open communication, family cohesion, and coping skills.	Cognitive-behavioural theory	**Psycho-educative elements in 4** modules**:** (1) Understanding the diagnosis (2) Coping strategies (3) Family communication (4) Long-term adjustment	1. 4 weeks each 2. No fixed schedule 3. 16 weeks	1. Web-based platform 2. No information 3. Family-based. 4. Psychologist 5. Home	0	**Anxiety and depression:** HADS **Partnership quality:** PFB **Family functioning:** FACES-IV	Mother reported improved family cohesion and reduced anxiety; children used age-appropriate tools	NA
Yu Xihong et al. (2018) China	Qualitative evaluation study	10 breast cancer patients (aged 25–45, raising children aged 6–12)	Communication education programme	Improve the communication skills of mothers with breast cancer, enhance parent-child relationships, assist children in understanding their mothers' illnesses, and alleviate their psychological stress.	None	Psycho-educative and communication skill-trained elements: (1) Inform children about the reasons and benefits of their mother's cancer diagnosis; (2) How to communicate with children about cancer (attitude, time, place, method); (3) Understand the possible emotional reactions of children (such as anxiety, fear, etc.); (4) Help children cope (by consulting books, doctors, or teachers); (5) Explain the process of cancer treatment and family changes; Listen to your children's feelings and observe their coping mechanisms.	1. No information 2. No information 3. No information	1. Telephone 2. No information 3. Individually 4. Nurse and psychologist 5. Home	0	Semi-structured interview	The communication skills of mothers with breast cancer have significantly improved; Improved parent-child relationships lead to a more harmonious family atmosphere.	100%
McKinney (2018) USA	Study protocol pre-/ post intervention study	African American families (parent with cancer and adolescent children aged 12–18	Families Fighting cancer together (FFCT)	Reduce parental stress and depressive symptoms in adolescents.	Attachment theory, social Learning theory	**Culturally tailored, resilience, support and coping elements in 5** sessions**:** Sessions 1–2: Adolescent-only group sessions (90 min). Topics: Emotional awareness, coping skills, communication preparation. Session 3: Parent-only session (2 hrs). Topics: Self-care, validating adolescent emotions, Afro-centric resilience. Sessions 4–5: Multiple family group sessions (2 hrs). Topics: Open communication, shared experiences, strengthening attachment.	1. 90 min to 2 hours depending on the session. 2. Every two weeks 3. 8 weeks	1. Face to face 2. No information 3. In-person group sessions 4. therapists 5. Hospital	NA	**Depression:** CES-D **Anxiety:** STAI **Parenting concerns:** PCQ **Quality of life:** FACT-G	None, study protocol.	NA
Denzinger et al. (2019) Switzerland	RCT	22 families (34 parents, 29 children/adolescents), Intervention group: 15 families, Control group: 7 families	FAMOCA (family online counselling for families with parental cancer)	Improve psychological adjustment in families, enhance open communication, family cohesion, and coping skills.	Cognitive-behavioural theory	**Psycho-educative elements in 4** modules**:** (1) understanding the diagnosis (2) Coping strategies (3) Family communication (4) Long-term adjustment	1. 4 weeks each 2. No fixed schedule 3. 16 weeks	1. Web-based platform 2. No information 3. Family-based. 4. Psychologist 5. Home	54.00%	**Family functioning:** FACES IV	None, only provides baseline data.	60%
Khanjari et al. (2020) Iran	Pre-test/post-test design	70 mothers with breast cancer who had adolescent daughters aged 15–18 years old (35 in each of the control and education groups)	Coping skills training for Daughters of mothers with breast cancer	Enhance coping strategies to manage stress related to maternal breast cancer. Provide education about breast cancer, self-care, and emotional support mechanisms	No information	**Psycho-educative and skill training elements in 4** sessions**:** (1) Cancer education: Basics of cancer, staging, risk factors. (2) Emotional adaptation: Identifying emotions (fear, anger, sadness), family role changes, and coping strategies. (3) Self-care and prevention: Breast self-examination, healthy lifestyle factors. (4) Support systems: Identifying resources (family, counselors, support groups).	1. 1.75–2 hours 2. No information 3. 4 weeks	1. Face to face 2. Booklets, pamphlets, and moulage for breast self-exam practice. 3. In-person workshops 4. Team: A child/adolescent psychiatrist, two pediatric nurses, and a psychiatric nursing professional. 5. Hospital	7.90%	Coping strategies of patients	Mothers reported improved the coping strategies (self-care, communication with child and emotional support).	80%
Konings et al. (2020) Australia	Post intervention study	19 parents (16 patients, 3 partners)	Parenting through cancer – A guide for parents of adolescents and young adults dealing with cancer in the family	Provide evidence-based information to parents with cancer who have adolescent. Normalize parental concerns (e.g., communication, financial stress, child support). Facilitate discussions between parents and healthcare professionals (HCPs)	No information	**Psycho-educative and skill training elements in 4** topics**:** (1) Impact of cancer on family/parenting. (2) Communication strategies (e.g., breaking news, ongoing discussions). (3) Children's reactions and support strategies. (4) Financial assistance, self-care, and additional resources.	1. No information 2. Single delivery 3 .No information	1. Online 2. Booklet distributed by HCPs or downloaded online 3. Self-guided 4. Oncologists, nurses, social workers 5. Clinical settings (e.g., hospitals, oncology clinics) and online.	NA	Interview	Increased confidence in communication, child support, and emotional management.	NA
Lewis et al. (2020) USA	Pre-/post intervention study	16 parents with children (5–17 years)	Enhancing connections-group	(1) Address the communication and parenting issues. (2) Decrease maternal depressed mood and anxiety, improve parenting behavior (parenting quality, skills and self-efficacy), and (3) improve children's behavioral-emotional adjustment to their mother's breast cancer.	The transtheoretical model of coping; the contextual model of parenting; Bandura's social cognitive theory	**Psycho-educative, reflective and skill- and efficacy-building** elements **in 5 sessions** (1) Assist mothers in managing cancer-related emotions and enhancing self-care skills; (2) Increase listening skills; (3) Application of listening skills; (4) Evaluate a child's coping strategies and measures that can be taken to cope with cancer-related stress; (5) Self-reflection exercises help mothers build confidence.	1. 1 hour 2. No information 3. No information	1. Fully manualized, group-delivered 2. A parent workbook, and handouts. 3. Family-centered fully manualized, group-delivered cancer parenting education 4. Nurse navigators, clinical trial nurses, social workers 5. No information	28.57%	**Mother's anxiety:** STAI: 20 **Mother's depression:** CES-D: 20 **Parenting self-efficacy:** CASE **Parenting skills:** PSC	Parent's depressed mood & anxiety ↓ (*P* = 0.024) Parenting self-efficacy ↑ (*P* < 0.01) Patient's parenting skills ↑ (*P* < 0.01) Child's behavioural-emotional adjustment →	80%
Zahlis (2020) USA	Qualitative study	16 parents with children (5–17 years)	Enhancing connections-group	(1) Address the communication and parenting issues. (2) Decrease maternal depressed mood and anxiety, improve parenting behavior (parenting quality, skills and self-efficacy), and (3) improve children's behavioral-emotional adjustment to their mother's breast cancer.	The transtheoretical model of coping; the contextual model of parenting; Bandura's social cognitive theory	**Psycho-educative, reflective and skill- and efficacy-building elements in 5** sessions (1) Assist mothers in managing cancer-related emotions and enhancing self-care skills; (2) Increase listening skills; (3) Application of listening skills; (4) Evaluate a child's coping strategies and measures that can be taken to cope with cancer-related stress; (5) Self-reflection exercises help mothers build confidence.	1. 1 hour 2. No information 3. No information	1. Fully manualized, group-delivered 2. A parent workbook, and handouts. 3. Family-centered fully manualized, group-delivered cancer parenting education 4. Nurse navigators, clinical trial nurses, social workers 5. No information	28.57	What they had gained or thought about as a result of participating in “enhancing connections Programme”. Semi-structured interviews.	(1) Being ready for a conversation about my cancer; (2) Bringing things out in the open; (3) Listening better to my child; (4) Getting my child to open up; (5) Not getting in my child's way; (6) Changing my parenting.	100%
Alvariza et al. (2021) Sweden	Pre-/post intervention study	20 families (29 parents: 13 ill parents, 16 co-parents; 9 mothers and 11 fathers with life-threatening illnesses, mean age 48 years; 19 co-parents)	The family talk intervention (FTI)	Increase family communication about illness-related consequences and support parenting. Promote open, honest family communication. Improve understanding of the disease among family members. Support children's needs and resilience.	No information	**Psycho-educative, emotion regulation and discussion in 6 Meetings:** Meetings 1–2: Parents discuss illness impact, children's needs, and set goals. Meeting 3: Individual child sessions (without parents) to explore feelings and questions. Meeting 4: Parents plan the family meeting based on children's input. Meeting 5: Family meeting led by parents to discuss shared concerns. Meeting 6: Follow-up to address ongoing needs.	1. Not explicitly stated 2. Meetings held 1–2 weeks apart. 3. Approximately 6–12 weeks	1. Face to face 2. No information 3. Family-centered 4. Two trained professionals (a deacon and a medical social worker) 5. Home	10.00%	Study-specific questionnaires developed by researchers for ill parent, the co-parent, and the children/baseline (questionnaire), upon intervention completion (questionnaire and interviews), and1 year after baseline (questionnaire)	Parents reported improved family communication. Parents feeling understood by the interventionists, and a majority (86%) felt at ease with sharing their thoughts and feelings freely during the meetings.	80%
Stafford et al. (2021) Australia	Pre-/post intervention study	12 cancer patients, 5 co-parent	“Enhancing parenting in cancer (EPIC) "	(1) Improve parenting efficacy and promote family communication (2) Decreasing parental stress and psychological morbidity (3) Enhancing children's psychosocial adjustment	No information	**Psycho-educative, and skill- elements 3** modules**:** (1) **AV**R: This 1-h, psychoeducational chaptered tool covered; communicating well with children; talking about diagnosis and treatment; talking with preschoolers/primary schoolers; conversations about the future and fears of death; maintaining family routine and making the most of supports. (2) QPL: This suggested 19 questions to ask HPs to bridge any gaps between general AVR information and information specific to individuals, including how diagnosis, treatment or side-effects may impact parenting. (3) Follow-up call: Conducted by a psychologist to review learnings from the AVR and QPL, discuss specific issues concerning parents/ partners/children and provide referrals/additional resources on targeted topics (e.g., managing hospital visits), if required.	1. 1-h AVR; QPL and follow-up call is variable 2. No information 3. No information	1. Web-based delivery; Phone 2. AVR 3. Parent-centered intervention 4. Psychologist 5. Home	34.60%	**Quality of life:** FACT-G **Family functioning:** FAD **Parental psychological morbidity:** DASS-21 **Parenting stress:** PSI-R SF **Parenting self-efficacy and satisfaction:** PSOCS **Parenting concerns:** PCQ **Parental perceptions of the behavioral functioning of child:** SDQ	Quality of life ↑ Family functioning ↑ Parental psychological morbidity ↓ Parenting stress ↓ Parenting self-efficacy and satisfaction ↑ Parenting concerns ↓ Parental perceptions of the behavioural functioning of children ↑	60%
Sun (2021) China	RCT	54 breast cancer patients (Intervention: 28; Control: 26)	Breast cancer patients and their minor Children's disease communication intervention program	Reduce anxiety and depression in patients and their children. Improve family functioning. Promote effective parent-child communication about the disease.	Family systems theory	**Psycho-educative, and skill- elements 3** sessions: (1) health education on breast cancer related knowledge and issue relevant health education materials (2) A health education on emotional management and parent-child disease notification strategies. (3) A health education on parent-child interaction strategies	1. 20–40 minutes 2. The first day after admission, before discharge, and one week after discharge 3. No information	1. Face to face combined with WeChat platform 2. Health education manuals 3. Mother only 4. Nurse 5. Hospital and home	7.40%	**Mother's anxiety:** S-AI **Family functioning:** FAD	Effectively improved emotional well-being (interaction effect: *F* = 3.515, *P* < 0.05) and family function (*P* < 0.05)	60%
Dohmen et al. (2021) Germany	Study protocol mixed-methods quasi-experiment study	Planned: 560 families with parental cancer	Family-SCOUT	Provide support for families with minors suffering from parental cancer	The COSIP (children of somatically ill parents) manual	**Psycho-education, emotion regulation and supports in 4 modules:** (1) Needs assessment: Initial evaluation of family-specific psychosocial needs. (2) Organizational support (e.g., establishing household help or advising on securing finances) (3) Communicative support (e.g., providing age-appropriate information material on cancer for the children) (4) Emotional support (e.g., developing functional coping strategies).	1. Initial interview: 2 hours. Home Visits: 1–2 hours. Video calls: 1 hour. Telephone calls: 20–40 minutes. 2. Flexible; typically 1 home visit/month 3. 9 months	1. Home visits, telephone support, text/email messages, or video calls 2. No information 3. No information 4. Social workers collaborated with healthcare providers, schools, youth welfare, and therapists. 5. Hospital, home, community	None, study protocol.	NA	None, study protocol.	NA
Lewis et al. (2021) USA	Pre-/post intervention study	15 mothers with various cancer types and 1 spouse	Enhancing connections-group (EC-G)	Decrease maternal depressed mood and anxiety, improve parenting behaviour (parenting quality, skills and selfefficacy), and improve children's behavioural emotional adjustment to their mother's breast cancer	A developmental-contextual model of parenting, the transtheoretical model of coping, and Bandura's social cognitive theory	**Psycho-educative, reflective and skill- and efficacy-building elements in 5** sessions (1) Anchoring yourself to help your child: Managing parental emotions. (2) Adding to your listening skills: Introducing the listening Framework. (3) Building on your listening skills: Eliciting child's concerns. (4) Being a Detective of your Child's coping: Identifying child's coping strategies. (5) Celebrating your success: Reflecting on gained skills.	1. 1.5 hours 2. 2-week intervals 3. 10 weeks	1. Face-to-face 2. No information 3. Group intervention 4. Group facilitator 5. Community	23.80%	**Depressed mood:** CES-D **Anxiety:** STAI **Parenting self-efficacy:** CASE **Parenting quality:** FPRQ **Parenting skills:** PSC	Parents' scores on depressed mood and anxiety decreased between baseline and postintervention assessments and parents' anxiety changed but the change was not significant for parents' depressed mood. Parenting self-efficacy improved between baseline and post-intervention. Parents' scores on parenting quality and parenting skills improved.	80%
Falk et al. (2022) Sweden	Pre-/post intervention study	20 families (39 parents; children aged 6–19)	The family talk intervention (FTI)	Increase family communication about illness-related consequences and support parenting. Promote open, honest family communication. Improve understanding of the disease among family members. Support children's needs and resilience.	Psycho-education, narrative theory and dialogical theory	**Psycho-educative, emotion regulation and discussion in 6 Meetings:** Meetings 1–2: Parents discuss illness impact, children's needs, and set goals. Meeting 3: Individual child sessions (without parents) to explore feelings and questions. Meeting 4: Parents plan the family meeting based on children's input. Meeting 5: Family meeting led by parents to discuss shared concerns. Meeting 6: Follow-up to address ongoing needs.	1. Not explicitly stated 2. Meetings held 1–2 weeks apart. 3. Approximately 6–12 weeks	1. Face to face 2. No information 3. Family-centered 4. Two trained professionals (a deacon and a medical social worker) 5. Home	15.00%	Study-specific questionnaires (Likert-scale items) on family communication, relationships, and worry. Semi-structured interviews.	Parents reported enhanced open dialogue and understanding of perspectives. Strategies learned (e.g., one-on-one time) improved parent-child/partner bonds. Ill parents felt secure about their family's future.	80%
Eklund et al. (2022) Sweden	Pre-/post intervention study	7 families (7 parents with cancer, 8 partners and 16 children)	The family talk intervention (FTI)	Increase family communication about illness-related consequences and support parenting. Promote open, honest family communication. Improve understanding of the disease among family members. Support children's needs and resilience.	Psycho-education, narrative theory and dialogical theory	**Psycho-educative, emotion regulation and discussion in 6 Meetings:** Meetings 1–2: Parents discuss illness impact, children's needs, and set goals. Meeting 3: Individual child sessions (without parents) to explore feelings and questions. Meeting 4: Parents plan the family meeting based on children's input. Meeting 5: Family meeting led by parents to discuss shared concerns. Meeting 6: Follow-up to address ongoing needs.	1. No information 2. Every 2–3 weeks 3. Varied from 6 to 19 weeks	1. Face-to-face individually and with the family 2. No information 3. Family-centered 4. Two trained professionals (a deacon and a medical social worker) 5. Hospital	12.50%	Closed-ended questions about communication	FTI was perceived as a help to prepare for and talk about what was to come, which mainly focused on promoting open communication. The parents especially emphasised the importance of communicating with the children (e.g. by helping them talk about their worries).	80%
Liénard et al. (2022) Germany	RCT	60 parents, 31 were intervention group and mean 44 ± 6 years, and 29 was control group were 41 ± 7 years, both the designated child was 11 ± 4 years	Parental support intervention	Improve parents' communication self-efficacy and perceived communication behaviors with children. Decrease parenting concerns and distress.	No information	**Psycho-educative, skills training and support elements in 4** sessions**:** (1) Assessment of family context and communication challenges. (2) Understanding children's reactions/needs. (3) Skill-building (e.g., role-playing, relaxation). (4) Reinforcement of communication strategies.	1. 1.5 hours/session 2. weekly 3. 7 weeks	1. Face to face or telehealth 2. Informational booklet 3. Family-centered 4. Psychologist 5. Home or hospital	9.00%	**Communication self-efficacy**:16-item scale. Perceived communication behaviors (broadness, calmness). **Parenting concerns:** PCQ **Distress:** HADS.	Increased communication self-efficacy, reduced communication difficulties, and increased the knowledge about how to communicate. No significant changes in parental distress or parenting concerns.	80%
Erhbar et al. (2022) Switzerland	Pre-/post intervention study	10 families (9 mothers with breast cancer, 8 partners and 12 children of unknown age)	A short-term counselling intervention	Enhance family communication and cohesion. Reduce anxiety and depression levels in parents. Improve quality of life for children and adolescents. Support parental partnership quality.	The COSIP (children of somatically ill parents) manual	**Psycho-education, emotion regulation and assignments in** six **sessions:** (1) Evaluation: Assessment of family situation. (2) Diagnostic feedback: Analysis of baseline questionnaires and family needs. (3) Detecting family resources (e.g., skill-sharing activities). (4) Managing everyday life (e.g., creating a family calendar). (5) Dealing with emotions (e.g., mindfulness exercises). (6) Closing session: Reflection on progress and consolidation of strategies.	1. No information 2. No information 3. 6 weeks	1. Face-to-face counselling sessions 2. NR 3. NR 4. Interdisciplinary team (psychologists, gynecologists, oncologists) 5. Hospital	30%	**Family communication:** FACES **Parental anxiety and depression:** HADS **Quality of relationship:** German questionnaire “partnerschaftsfragebogen” (marital quality ques-tionnaire).	Mothers showed an increase from pre to post intervention regarding communication and satisfaction. Mother's anxiety and depression showed no significant differences from pre-to post-intervention. Quality of relationship with partner had no difference pre-to post- intervention.	80%
Park et al. (2022) USA	Pre-/post intervention study	46 parents with various types of cancer	Families Addressing cancer together (FACT)	To address the communication Needs of parents with cancer	The health Disclosure Decision-making model and social cognitive theory	**Psycho-education and skill building elements in 5** modules**:** (1) **Introduction:** General principles of parental communication about cancer. (2) First conversations: Guidance on discussing a new diagnosis or recurrence, including scripts for describing cancer, treatment, and reassurance. (3) Follow-up conversations: Strategies for ongoing discussions, child reactions, and nonverbal communication. (4) Common questions and concerns: Optional topics (e.g., answering questions about death, handling emotional reactions). (5) Additional resources: Curated links and materials for families.	1. Not explicitly stated, but the intervention is self-administered online, allowing flexibility. Parents could view all modules at once or incrementally. 2. Single delivery at baseline, with no additional sessions mentioned. 3. No information	1. Online individually 2. Self-guided, web-based system 3. Parents received the intervention once, tailored to one child (though they could involve co-parents) 4. Fully automated 5. Home	46%	**Communication self-efficacy:** CSES **Communication behaviors/beliefs:** PCCQ **Mental health:** HADS **Quality of life:** FACT-G **Family functioning:** GFS	Two-weeks post intervention, parents reported stable-to improved scores on confidence for talking about their illness in an age appropriate way; coming up with a plan for how to tell their child and handling their child's emotional response. There were no significant changes in HADS, FACT-G, or GFS scores from pre- to 2- or 12-weeks post-intervention. Parents felt that the intervention helped them feel more comfortable and prepared to talk with their children about their illness.	60%
Melchiors et al. (2022) Germany	Qualitative evaluation study	9 parents (patients with various cancer types and partners)	Information booklet	Address a lack of information on age specific communication and developmental aspects of children and an overview of local support offers for affected families	No information	**Psycho-education in 5** modules**:** (1) Communication about cancer and emotions within the family. (2) Living with cancer and establishing a “new normal” family life. (3) Age-specific reactions of children to parental cancer. (4) Addressing disease progression and loss. (5) Local support services and additional resources.	1. No information 2. No information 3. No information	1. Text-based: Booklet 2. Printed booklet (40 pages) 3. Parents single 4. No information 5. Clinical and community settings	18.18%	Interviews/the acceptability and usability of the booklet and information needs were conducted about 1 week after receiving the booklet	The useful booklet should be handed out prosonally, and parents need information: (a) Communication, (b) support offers,(c) children's disease understand ingand needs, (d) organization of family life, (e) competence in parenting, and (f) sources of additional information material	80%
Phillips et al. (2022) USA	RCT	50 parent-child dyads with various types of parental cancer, Intervention group: 28 families, Control group: 22 families	Wonders & Worries advanced cancer (WW-AC)	Improve family quality of life, functioning, and communication. Enhance children's emotional/behavioral adjustment and parenting efficacy. Reduce parenting concerns and ill parents' depression/anxiety.	The resiliency model of family stress, adjustment and adaptation	**Psycho-educative, emotional expression, and coping skills in 9 sessions:** **Parent consultations (2 sessions):** Discuss child's understanding of cancer, coping skills, and family communication strategies. **Child sessions (6 weekly individual sessions):** Session 1: Rapport-building (games, art activities). Session 2: Cancer education (worry box, medical equipment demonstrations). Session 3: Emotional expression (feelings bingo, art). Sessions 4–5: Stress/coping skills (coping kits, relaxation techniques). Session 6: Closure/hopes for the future (art activities). **Treatment center tour (1 session):** Demystify medical environments for children.	1. 1 hour 2. Weekly 3. 6 weeks (child sessions) + 2 parent consults + 1 tour.	1. Face-to-face in the family 2. No information 3. No information 4. A child life specialists (CCLS) 5. Community	16.7%	**Parenting concerns:** PCQ **Parenting self-efficacy:** CASE **Depressed Mood:** CES-D-R **Anxiety:** STAI	Parenting concerns were significantly lower at 6 weeks (Cohen's d = 0.95), and at 10 weeks (Cohen's d = 0.70) in the intervention group compared with the control group. Parents in the intervention group at 6 weeks were significantly more confident in their ability to help the child deal with cancer-related concerns (Cohen's d = 1.0), better able to deal and manage the demands of having cancer (Cohen's d = 0.94), and able to stay calm to a greater extent while interacting with the child about cancer (Cohen's d = 0.59). Parents in the intervention group reported higher emotional well-being at 6 weeks compared with the parents in the control group (Cohen's d = 0.89). There were no significant differences between the intervention and control groups on the parent Depressed Mood & anxiety or on the FAD communication subscle at either six or 10 weeks.	80%
Palacios et al. (2023) USA	Pre-/post intervention study	18 mothers with non-metastatic cancer and 18 children	Conexiones (a culturally adapted cancer parenting education program for diagnosed child-rearing hispanic mothers)	To culturally adapt enhancing connections (EC) and decrease maternal depressed mood and anxiety, improve parenting behaviour (parenting quality, skills and self-efficacy), and improve children's behavioural emotional adjustment to their mother's breast cancer	Collins' developmentalcontextual model of parenting and Bandura's social cognitive theory	**Psycho-educative, reflective and skill- and efficacy-building elements in 5** sessions: (1) Anchoring yourself to help your child: Managing parental emotions. (2) Adding to your listening skills: Introducing the listening Framework. (3) Building on your listening skills: Eliciting child's concerns. (4) Being a Detective of your Child's coping: Identifying child's coping strategies. (5) Celebrating your success: Reflecting on gained skills.	1. 1.5 hours 2. 2-week intervals 3. 10 weeks	1. Telephone 2. No information 3. Group intervention 4. Group facilitator 5. Community	0	**Depressed mood:** CES-D **Anxiety:** STAI **Parenting self-efficacy:** CASE **Parenting quality:** FPRQ **Parenting skills:** PSC	Mothers' s cores on depressed mood decreased significantly between baseline and post-intervention. Scores on anxiety decreased between baseline and post-intervention, but the change was not statistically significant. Scores on parenting self-efficacy significantly improved. Scores on parenting quality improved.	60%
Plont et al. (2023) Denmark	Qualitative design (4–8 weeks post-interventin)	5 women with breast cancer and children aged 5–16 years	The Children's tour	Reduce distress and enhance the feeling of security within families affected by breast cancer. Support communication between parents and children regarding cancer-related matters	No information	**Psycho-educative, discussion and efficacy-building elements in 5** modules**:** (1) Tour of the chemotherapy treatment room. (2) Hands-on interaction with medical equipment (e.g., syringes, treatment chairs). (3) Group discussion about cancer and chemotherapy led by nurses. (4) Watching an educational film featuring a familiar actor. (5) Refreshments and a teddy bear gift for children.	1. No information 2. 4 times a year 3.1 hour 15 minutes.	1. In-person, group-based sessions 2. No information 3. No information 4. Nurse 5. Hospital	0	A semi-structured interview	Parents felt more confident discussing cancer and chemotherapy with their children.	60%
Ann-Yi et al. (2023) USA	Pre-/post intervention study	10 dyads (10 patients + 10 spouses)	Parent support program for dual caregivers	Reduce parenting concerns and improve parenting efficacy in metastatic cancer patients and their spouses. Alleviate psychological distress (anxiety/depression) related to dual caregiving roles. Enhance family communication and legacy-building for children.	Cognitive social theory	**Psycho-educative, emotional expression, and skill- elements in 4-session:** **Dyadic sessions (patient + spouse, 2 sessions):** Session 1: Illness communication (child's understanding, age-appropriate language). Session 2: Family routines/legacy-making (maintaining rituals, post-death connections). **Individual spouse sessions (2 sessions):** Session 3: Caregiver coping (self-care, social support, guilt/anxiety management). Session 4: End-of-life preparation (child grief, funeral planning).	1. 45 minutes 2. Weekly 3. 4 weeks	1 Video conferencing (Zoom). 2. No information 3. In-person, dyadic sessions 4. Licensed psychological counselor (LPC) 5. Remote delivery via zoom	5%	**Psychological symptoms:** HADS **Parenting concerns:** PCQ **Parenting efficacy:** CaPSE **Relationship satisfaction:** DAS-7	Patients: Reduced parenting concerns (*P* = 0.003), improved efficacy (*P* = 0.03) at 6 weeks. Spouses: Reduced depression (*P* = 0.04), improved efficacy (*P* < 0.001) at 6 weeks; sustained concern reduction at 12 weeks (*P* = 0.001).	60%
Akagawa et al. (2023) Japan	Mixed-methods (quantitative pre-post intervention + qualitative interviews)	5 parents	Children's lives include Moments of bravery (CLIMB®) program.	Improve parents' quality of life (QoL) and reduce psychological anxiety. Enhance parent-child communication about cancer. Empower children to cope with parental illness. Reduce parental guilt and isolation.	Intrinsic Motivation theory, social support theory	**Psycho-education, emotion regulation, and discussion elements in 4 sessions:** Parents' group: Open discussions with healthcare providers about parenting challenges, treatment, and child communication. Children's Group: Session 1: Sharing cancer stories, crafting “happy/sad” masks. Session 2: Cancer education (e.g., treatment effects, debunking myths). Session 3: “Strong box” activity for anxiety management. Session 4: Anger management cube and “caring cards” for parent-child communication.	1. 2.5 hours 2. No information 3. No information	1. In-person, group-based 2. Crafts, educational tools 3. No information 4. Nurses, psychologists 5. Hospital	0%	**Quality of life:** FACT-G **Anxiety:** STAI Semi-structured interviews on parental emotional changes and child behaviors.	Significant improvement in emotional subscale scores (*P* = 0.04). Significant reduction in state (*P* = 0.04) and trait anxiety (*P* = 0.04). Parents reported reduced guilt and improved self-acceptance. Strengthened family cohesion and coping.	80%
Ernstmann et al. (2024) Germany	A mixed-methods quasi-experiment study	472 families with parental cancer, Intervention group: 262 families, Control group: 210 families	Family-SCOUT (comprehensive psychosocial intervention for families with parental cancer)	Reduce psychological burden on families coping with parental cancer. Strengthen family resilience through tailored support. Provide cross-sectoral, family-centered care (e.g., emotional, communicative, and organizational support).	The COSIP (children of somatically ill parents) manual	**Psycho-education, emotion regulation and supports in 4** modules**:** (1) Needs assessment: Initial evaluation of family-specific psychosocial needs. (2) Organizational support (e.g., establishing household help or advising on securing finances) (3) Communicative support (e.g., providing age-appropriate information material on cancer for the children) (4) Emotional support (e.g., developing functional coping strategies).	1. Initial interview: 2 hours. Home Visits: 1–2 hours. Video calls: 1 hour. Telephone calls: 20–40 minutes. 2. Flexible; typically 1 home visit/month 3. 9 months	1. Home visits, telephone support, text/email messages, or video calls 2. No information 3. No information 4. Social workers collaborated with healthcare providers, schools, youth welfare, and therapists. 5. Hospital, home, community	0.00%	6 semi-structured interviews	Participants reported perceived emotional, communicative, and organizational support.	80%
Meyer et al. (2024) Germany	A mixed-methods quasi-experiment study	472 families with parental cancer, Intervention group: 262 families, Control group: 210 families	Family-SCOUT (comprehensive psychosocial intervention for families with parental cancer)	Reduce psychosocial distress in parents (both sick and healthy) of families affected by cancer. Provide structured, cross-sectoral support to maintain daily life, improve family communication, and enhance emotional coping. Address unmet needs of all family members (parents and children) across disease phases, including palliative care and bereavement.	The COSIP (children of somatically ill parents) manual	**Psycho-education, emotion regulation and supports in 4** modules**:** (1) Needs assessment: Initial evaluation of family-specific psychosocial needs. (2) Organizational support (e.g., establishing household help or advising on securing finances) (3) Communicative support (e.g., providing age-appropriate information material on cancer for the children) (4) Emotional support (e.g., developing functional coping strategies).	1. Initial interview: 2 hours. Home Visits: 1–2 hours. Video calls: 1 hour. Telephone calls: 20–40 minutes. 2. Flexible; typically 1 home visit/month 3. 9 months	1. Home visits, telephone support, text/email messages, or video calls 2. No information 3. No information 4. Social workers collaborated with healthcare providers, schools, youth welfare, and therapists. 5. Hospital, home, community	26.8%	**Psychosocial distress:** HADS	The intervention was associated with a significant reduction in parental distress in the intervention group (MID 70.4% in at least one parent) compared with the control group (MID 55.8%; *P* = 0.008)	100%
Milbury et al. (2025) USA	RCT	50 dyads (patients with metastatic cancer and their spousal caregivers)	Parent support intervention	Address unmet parenting concerns and psychological distress in patients with advanced cancer and their spouses. Improve cancer-related parenting efficacy, illness communication, family routines, caregiver support. Reduce anxiety and depression symptoms in patients and caregivers.	Cognitive social theory	**Psycho-education, skill- and efficacy-building and supports elements in 4 sessions:** Session 1 (Dyadic): Developmentally appropriate communication about cancer with children. Session 2 (Dyadic): Maintaining/modifying family routines and creating a meaningful legacy. Session 3 (individual for spouses): Caregiver coping strategies and psychological support. Session 4 (individual for spouses): Preparing children for EOL (e.g., understanding death, grief resources).	1. 45 minutes 2. Once per week 3. 4 weeks	1. Video-conference (Zoom) 2. Workbook: Provided educational materials 3. Dyadic sessions and individual sessions. 4. Licensed professional counselors (LPCs) 5. Home-based (delivered remotely via zoom)	14%	**Psychological symptoms:** HADS **Parenting concerns:** PCQ **Parenting efficacy:** CaPSE	Multilevel analyses revealed a signifcant reduction in anxiety symptoms (*P* = 0.05) , parenting concern (*P* < 0.05) and improvement in parenting effcacy (*P* = 0.03) at 6-week follow-up in the intervention group compared with usual care.	60%
Ross et al. (2024) Australia	A mixed-methods design (pre-/post intervention study + qualitative study)	36 parents	Parent support worker (PSW) service	Provide integrated psychosocial support to parents with cancer, partners, and children. Address parenting concerns (communication, emotional coping, practical issues). Reduce psychological distress and improve parenting efficacy. Facilitate referrals to additional support services.	No information	**Psycho-education, skill-, counselling and supports elements in 4** modules**:** (1) Individualized support (e.g., communication strategies, emotional coping). (2) Referrals to psychologists, music therapy, etc. (3) Resource provision (e.g., educational materials, websites). (4) Flexible follow-ups (some parents desired more sessions).	1. Not explicitly stated, individually 2. Not explicitly stated, individually 3. Not explicitly stated, individually	1. Face to face or telephone 2. No information 3. Non-standardized: Tailored to individual family needs. 4. Social workers 5. Three Australian hospitals.	0	**Psychological distress:** The Kessler-10 (K10) **Parenting concerns:** PCQ **Parental efficacy:** CASE	Parents' distress, parenting concerns, parenting efficacy, and stress about situations of concern improved significantly from pre- to post-service (all *P* < 0.005) Interview: Improved confidence to communicate with and support children. Improved emotional coping	80%
Bergersen et al. (2024) Sweden	A qualitative study	9 parents (3 ill parents, 6 co-parents)	The family talk intervention (FTI)	To facilitate family communication about illness-related subjects. To support parenting and make children's psychosocial needs visible. To alleviate feelings of loneliness and provide long-term coping strategies.	Psycho-education, narrative theory and dialogical theory	**Psycho-educative, emotion regulation and discussion in 6 Meetings:** Meetings 1–2: Parents discuss illness impact, children's needs, and set goals. Meeting 3: Individual child sessions (without parents) to explore feelings and questions. Meeting 4: Parents plan the family meeting based on children's input. Meeting 5: Family meeting led by parents to discuss shared concerns. Meeting 6: Follow-up to address ongoing needs.	1. Not explicitly stated 2. Meetings held 1–2 weeks apart. 3. Approximately 6–12 weeks	1. Face to face 2. No information 3. Family-centered 4. Two trained professionals (a deacon and a medical social worker) 5 .Home	45%	Semi-structured interviews	FTI reduced feelings of loneliness and improved family communication. Parents valued professional support and peer networks formed during FTI.	100%
Kroll et al. (2025) USA	Pre-/post intervention study	46 dyads (patients with advanced breast cancer + partners)	Standardized spousal discussion task	Explore couples' cancer-related parenting communication behaviors and perceptions. Identify associations between communication, psychological distress, and relational wellbeing. Inform targeted interventions for parents with advanced cancer.	Dyadic coping and communication models.	**Emotion regulation, discussion and support elements in 3** modules**:** (1) Family concern inventory (FCI): Couples independently rated 15 cancer-related parenting/family concerns (e.g., fear of death, role changes). (2) Discussion task: Each partner selected one concern to discuss for 10 minutes, aiming to problem-solve. (3) Post-discussion evaluation: Participants rated self-disclosure, partner validation, affect, and discussion utility.	1. 20 minutes 2. Single session 3. 20 minutes	1. Online via zoom 2. No information 3. Couple 4. Self-administered with remote researcher oversight (no direct facilitator) 5. Outpatient oncology clinic	6.50%	**Psychological distress:** CES-D (depression), GAD-7 (anxiety). **Relationship satisfaction:** DAS **Parenting concerns:** PCQ	Patients rated the discussionas more helpful than partners (*t* = 2.3, *P* = 0.03). There was a small reduction in positive affect following discussion for partnersonly.	60%
Shibata et al. (2025) Japan	A qualitative study	16 parents along with 19 children (6–12 years)	Children's lives include Moments of Br avery (CLIMB®) program	Facilitate emotional expression and communication between parents and children. Evaluate the adapted 4-session CLIMB® format in a Japanese clinical setting.	Intrinsic Motivation theory, social support theory	**Psycho-education, emotion regulation, and discussion elements in 4 sessions:** Children's Group: Session 1: Sharing cancer stories, crafting “happy/sad” masks. Session 2: Cancer education (e.g., treatment effects, debunking myths). Session 3: “Strong box” activity for anxiety management. Session 4: Anger management cube and “caring cards” for parent-child communication. Parents' group: Open discussions with healthcare providers about parenting challenges, treatment, and child communication.	1. 2 hours 2. 1–3 weeks 3. No information	1. Group-support 2. Structured activities 3. Closed-group format 4. Multidisciplinary team (nurses, psychologists, students) 5. Outpatient clinic	30%	Questionnaire with 13 questions	Reduced parents' parenting anxiety	60%

IBQ, The Interaction Behavior Questionnaire; CES-D, The Center for Epidemiological Studies Depression Scale; CDI, the Children's Depression Inventory; RCMAS, The Revised Children's Manifest Anxiety Scale; EORTC QLQ-C30, the European Organization for Research and Treatment of Cancer Quality of Life Questionnaire; ILC, Inventory for Quality of Life in Children and Adolescents; PCQ, Parenting Concern Questionnaire; SDQ, Goodman's Strengths and Difficulties Questionnaire; CBCL, Child Behavior Checklist; FAD, Family Assessment Device; PSC, Parenting Skills Checklist; CASE, Cancer Self-efficacy Scale; STAI, Spielberger State-Trait Anxiety Inventory; POPM, Perception of Parenting Measure; S-AI, State-Anxiety Inventory; SEI, Self Esteem Inventory; FACT-G, Functional Assessment for Cancer Therapy-General; KINDL, Kinder Lebensqualit.t; BDI-II, Beck Depression Inventory, Second Edition; FPRQ, Family Peer–Relationship Scale; BSI, Brief Symptom Inventory; BDI, Beck Depression Inventory; SCL-90, Symptom Checklist-90; FACIT–Sp, Functional Assessment of Chronic Illness Therapy Spiritual; Well-Being; HADS, The Hospital Anxiety and Depression Scale; IES-R, The Impact of Event Scale – Revised; PANAS, The Positive and Negative Affect Schedule; RSQ, 14 Response to Stress Questionnaire; FES, Family Environment Scale; DAS, The Dyadic Adjustment Scale; PSI-R SF, Revised Parenting Stress Index Short Form; PSOCS, Parental Sense of Competence Scale; DASS-21, Depression Anxiety Stress Scale; CSS, Crisis Support Scale; MSPSS, Multidimensional Scale of Perceived Social Support; GHQ-12, General Health Questionnaire; QOLS-N, Quality of Life Scale; SEPTI, Self-Efficacy for Parenting Tasks Index; PFB, Partnership Questionnaire, conflict, intimacy, mutuality; FACES-IV, Family Adaption and Cohesion Scale; PSOCS, Parenting self-efficacy and satisfaction; PCCQ, The Parental Cancer Communication Questionnaire; GFS, General Functioning Scale; CaPSE, Cancer Parenting Self-Efficacy Scale; DAS-7, Dyadic Adjustment Scale; GAD-7, Generalized Anxiety Disorder-7.

In the 53 included studies, 33 unique interventions were described. Interventions that were described and evaluated in multiple studies were: The Enhancing Connections Programme (EC) (*n* = 8), The Family Talk Intervention (FTI) (*n* = 4), Family-SCOUT (*n* = 3), Family online counselling for families with parental cancer (FAMOCA) (*n* = 2), and Children's Lives Include Moments of Bravery (CLIMB®) (*n* = 2).

### Characteristics of participants

Of the 53 reviewed studies, 23 conducted the interventions solely for parent,^11^^,^^14^^,^^25^^,^^28^^,^^35^, ^36^, ^37^, ^38^, ^39^, ^40^, ^41^, ^42^, ^43^, ^44^, ^45^, ^46^, ^47^, ^48^, ^49^, ^50^, ^51^, ^52^ 11 for parent-child dyads,^53^, ^54^, ^55^, ^56^, ^57^, ^58^, ^59^, ^60^, ^61^, ^62^, ^63^ and 9 for ill parent-spouse-child triads.^24^^,^^64^, ^65^, ^66^, ^67^, ^68^, ^69^, ^70^, ^71^ Most of the reviewed studies included ethnically diverse samples, while one study only recruited African American as the participants.^56^ 16 studies specifically targeted breast cancer patients^11^^,^^24^^,^^35^^,^^36^^,^^42^^,^^52^^,^^57^^,^^60^^,^^61^^,^^64^^,^^70^^,^^72^, ^73^, ^74^, ^75^, ^76^ while the remaining 37 focused on patients with a range of cancer types. The majority of studies encompassed parents diagnosed with cancer at stages 0-III,^36^^,^^37^^,^^39^^,^^56^^,^^65^^,^^71^ with two studies focusing exclusively on individuals with advanced-stage cancer.^25^^,^^40^ Most patients were young adults, with a mean age ranging between 39.1 and 45.6 years. The children involved in these studies were aged between 3 and 18 years. Participant attrition rates varied significantly across studies, ranging from 0% to 69%.

### Intervention characteristics

The intervention characteristics of the included studies are shown in Table 1.

#### Aims of interventions

Interventions were frequently designed to address a range of challenges, including psychological distress in parents diagnosed with cancer,^14^^,^^36^^,^^37^^,^^62^^,^^77^^,^^78^ facilitating adaptive coping.^73^ Common objectives of these interventions involved enhancing psychosocial adjustment to cancer among parents and families,^11^^,^^61^^,^^64^ improving psychological well-being^11^^,^^38^, ^39^, ^40^^,^^74^^,^^77^, ^78^, ^79^, and strengthening parent–child relationships.^53^^,^^80^ Additional goals included supporting parenting capabilities—such as fostering self-efficacy and improving parents’ ability,^11^^,^^15^^,^^35^^,^^37^^,^^39^^,^^40^^,^^57^^,^^66^^,^^67^^,^^73^^,^^74^^,^^79^^,^^81^ promoting cancer-related communication within the family/parent-child dyad^11^^,^^24^^,^^28^^,^^44^^,^^48^^,^^51^^,^^58^^,^^59^^,^^62^^,^^63^^,^^68^^,^^74^^,^^77^^,^^80^ and offering culturally sensitive psychosocial support.^50^^,^^56^

#### Theoretical bases of the interventions

The interventions in 30 studies were grounded in established theoretical frameworks, providing a structured foundation for their design and implementation. These frameworks included Preventive Intervention Models and Clarke's School-Age Child Support Group Model,^56^ the Family Resilience Theory,^73^ and Stress and Coping Theory.^66^^,^^67^ Other studies utilized Family Systems Theory combined with Developmental and Attachment Theories,^55^ as well as the Transtheoretical Model of Coping, the Contextual Model of Parenting, and Bandura's Social Cognitive Theory.^39^^,^^81^ These theoretical frameworks informed the intervention designs, ensuring they addressed key aspects of psychological and social functioning relevant to cancer-affected families.

#### Cultural setting of interventions

Among the 53 studies, one study was specifically tailored to the cultural context of the target population. Davey et al.^56^ developed a manualized, culturally adapted family intervention program specifically for African American families, delivered by an African American female therapist. This intervention demonstrated significant treatment effects.

#### Intervention content

Psychoeducation, parent-child communication guidance, social support, and self-efficacy training were common components across both parent- and family-focused interventions. Psychoeducation primarily involved helping patients identify and manage negative emotions such as anxiety and depression, teaching methods for emotional relief, and providing strategies for managing their own and their children's emotions to enhance support;^39^^,^^67^^,^^79^ These interventions often included several sessions with different topics and homework (e.g., booklets to read) for the parents and children. Topics included, for example, how to deal with emotions during information giving,^54^ building on parenting skills,^81^ adaptive coping with parental cancer,^56^ and how to maintain family functioning.^82^ Parent-child communication guidance focused on teaching patients how and when to disclose their illness to their underage children, along with strategies and skills for discussing disease-related matters effectively.^39^^,^^67^^,^^79^ Social support interventions aimed to mobilize resources from patients' families and communities to provide both direct and indirect assistance. Direct support included family meetings^56^ and psychological education.^36^ Indirect support guided patients in utilizing community resources, such as relatives, community networks, and churches, for emotional and spiritual reinforcement.^54^^,^^73^ Self-efficacy training sought to empower patients by recognizing and reinforcing their parenting capabilities through structured educational interventions, enabling them to feel more confident and effective in their parenting role.^39^^,^^67^

#### Intervention dosage

The dosage of an intervention encompasses its amount, frequency, and duration. The amount refers to the number of sessions, frequency indicates how often the intervention is delivered within a specific time frame, and duration represents the total length of time the intervention spans.^83^ These three aspects collectively define the dosage of the interventions included in this review. In the 53 studies, most interventions involve 2–6 sessions, and average time for per session ranged from 20 minutes to 2 hours. Intervention frequency varied widely, ranging from biannual or monthly sessions.^56^ In some studies, frequency was tailored to individual needs, following a needs-based approach.^55^^,^^66^ The length of individual sessions typically ranged between 1 and 2 hours. The total duration of interventions across the included studies ranged from as brief as 2 hours to as extensive as 12 months, reflecting the diversity in intervention design and delivery.

#### Intervention facilitators

All 53 included studies reported information regarding intervention facilitators. Twenty-three interventions were delivered by multidisciplinary teams comprising healthcare professionals such as nurses, social workers, and psychologists.^15^^,^^24^^,^^28^^,^^40^, ^41^, ^42^, ^43^^,^^45^^,^^51^^,^^52^^,^^54^^,^^55^^,^^58^^,^^59^^,^^63^^,^^69^^,^^70^^,^^72^^,^^73^^,^^76^^,^^77^^,^^81^^,^^84^ Sixteen interventions were led by a single professional group, most commonly clinicians, psychologists, or therapists.^11^^,^^14^^,^^27^^,^^36^^,^^48^^,^^50^^,^^53^^,^^56^^,^^57^^,^^60^^,^^61^^,^^64^^,^^67^^,^^68^^,^^74^^,^^85^ The remaining interventions were facilitated by nurses, trained counsellors, or health educators.^37^, ^38^, ^39^^,^^54^^,^^66^^,^^78^ Three studies did not provide detailed descriptions of the intervention facilitators.^35^^,^^37^^,^^47^ In addition, two interventions were self-administered by patients without direct professional facilitation.^25^^,^^75^

#### Delivery modes and formats

The interventions were delivered through a range of modalities. Twenty-two studies employed face-to-face delivery,^28^^,^^35^, ^36^, ^37^^,^^40^^,^^43^^,^^51^^,^^54^^,^^56^, ^57^, ^58^, ^59^, ^60^^,^^62^^,^^66^, ^67^, ^68^, ^69^^,^^73^^,^^77^^,^^80^^,^^85^ while three studies used telephone-based delivery.^38^^,^^45^^,^^79^ Six studies implemented online or web-based formats.^11^^,^^24^^,^^42^^,^^46^^,^^61^^,^^64^ In addition, 12 studies adopted a mixed-mode approach,^14^^,^^15^^,^^24^^,^^39^, ^40^, ^41^^,^^44^^,^^47^^,^^50^^,^^70^^,^^74^^,^^78^ typically combining face-to-face sessions with telephone follow-up or telehealth support. Three studies^53^^,^^65^^,^^86^ did not clearly specify the mode of delivery.

#### Intervention delivery settings

Interventions were delivered across a range of settings. Eighteen studies implemented interventions in participants’ homes,^11^^,^^27^^,^^37^, ^38^, ^39^^,^^43^^,^^45^^,^^51^^,^^53^^,^^54^^,^^56^^,^^58^^,^^61^^,^^64^^,^^74^^,^^75^^,^^79^^,^^80^ while two studies reported delivery in either home or hospital settings.^14^^,^^84^ Another 18 studies conducted interventions in hospitals or outpatient clinics.^25^^,^^28^^,^^35^^,^^42^^,^^50^^,^^52^^,^^59^^,^^60^^,^^62^^,^^63^^,^^67^, ^68^, ^69^^,^^72^^,^^73^^,^^77^^,^^78^^,^^85^ Eight studies adopted multi-setting approaches, delivering interventions across hospital, home, and community settings.^15^^,^^24^^,^^40^^,^^47^^,^^57^^,^^70^^,^^76^^,^^87^ Among the remaining studies, two reported flexible or participant-determined delivery settings,^36^^,^^66^ whereas several did not clearly specify the intervention setting.

### Intervention effectiveness

Among the included literature, a total of 45 scales or questionnaires were employed to assess the effects of psychosocial interventions on patients with cancer who parenting underage children. These measurement tools can be classified into the following six domains: psychological well-being, parenting behavior, self-efficacy, family dynamics, social behavior, and quality of life. Regarding assessment methodology, 16 studies primarily relied on a single interview method (e.g., structured, semi-structured, in-depth, or open-ended interviews) to evaluate the effectiveness of their interventions. An additional 3 studies adopted a combined approach, utilizing both scales/questionnaires and interviews for evaluation. A detailed description of the study outcomes and measurement instruments is provided in Table 1.

#### Psychological well-being

A total of 32 studies reported on the psychological well-being and functional status of participants, with key domains measured including anxiety, depression, psychological distress, illness-related fears, emotional functioning, and post-traumatic stress symptoms. Among them, 6 studies utilizing family-centered interventions^37^^,^^49^^,^^50^^,^^64^^,^^66^^,^^86^ and three studies employing parent-centered interventions^11^^,^^38^^,^^87^ reported statistically significant reductions in parental anxiety and depression, psychological distress, and illness-related fears (*P* < 0.05). One family-centered intervention study^67^ showed improved emotional functioning in parents compared to before the intervention (*P* < 0.05). However, in two other studies, no statistically significant changes in parental depression or anxiety levels were observed.^14^^,^^56^ Meanwhile, in 3 parent-centered interventions,^46^^,^^75^^,^^77^ no significant changes in parental anxiety and depression or post-traumatic stress symptoms were detected.

#### Parenting behavior

Among the included studies, 16 reported changes in parenting behaviors, including parenting actions, parenting skills, parenting concerns, and parenting quality. Among these, 5 studies on parent-centered interventions^11^^,^^35^^,^^38^^,^^39^^,^^53^ showed that the interventions significantly improved parenting behaviors, enhanced parenting skills and parenting quality, and reduced parenting concerns (*P* < 0.05). However, Lewis et al.^40^ noted that while mothers acquired and practiced new ways of communicating with their children, the quality of parenting itself did not change significantly. Meanwhile, 4 studies^40^^,^^41^^,^^49^^,^^50^ on family-centered interventions and 3 studies^40^^,^^54^^,^^76^ on interventions delivered by the clinical medical team in a home setting all reported that after the intervention, parents listened more attentively to their children, guided them to actively share their thoughts, refrained from excessive interference in their children's lives, adjusted their own parenting approaches, and showed significant improvement in parenting coping skills, parenting skills, and parenting quality (*P* < 0.05).

#### Self-efficacy

A total of 20 studies evaluated the impact of psychosocial interventions on the self-efficacy of parents, covering self-efficacy in coping with cancer, confidence in communicating about cancer, and parenting self-efficacy. Among these, 3 studies on family-centered interventions^14^^,^^50^^,^^73^ and 3 studies on parent-centered interventions^38^^,^^46^^,^^68^ reported positive effects in enhancing parents' confidence in coping with cancer and communication (*P* < 0.05), but provided limited evidence regarding the improvement of overall self-efficacy in coping with cancer. Additionally, 2 studies on parent-centered interventions^11^^,^^38^ and 3 studies on family-centered interventions^40^^,^^50^^,^^68^ indicated that psychosocial interventions effectively improved parents’ parenting self-efficacy and communication self-efficacy (*P* < 0.01).

#### Family dynamic

Among the included studies, 33 investigated how interventions affected family dynamics, with a focus on family functioning, family communication, family relationships, and family support. 6 studies on family-centered interventions^14^^,^^41^^,^^51^^,^^58^^,^^66^^,^^72^ indicated that family-centered interventions were notably effective in promoting positive interactions within the family, significantly improving family communication and enabling members to freely and easily share their thoughts and feelings. However, the results were not entirely consistent. 1 study^56^ found no significant changes in parent-adolescent relationships. 1 study^86^ reported an overall trend toward improved family functioning (*P* < 0.05); 2 studies^64^^,^^73^ noted enhanced family cohesion and support. Concurrently, 6 studies on parent-centered interventions^11^^,^^25^^,^^44^^,^^47^^,^^53^^,^^80^ also reported effective improvements in family relationships, family communication, and family functioning (*P* < 0.05).

#### Social behavior

A total of 7 studies highlighted the improvement in social behavior as a result of the interventions. This improvement was often observed alongside or in relation to changes in general parent-child communication, communication conflict behaviors, parent-child attachment, and the mitigation of social difficulties faced by parents due to cancer. 3 family-centered intervention studies^36^^,^^51^^,^^73^ demonstrated that psychosocial interventions facilitating the formation of social and peer networks could enhance the level of social support (*P* < 0.05).

#### Quality of life

Six parent-centered studies^11^^,^^28^^,^^46^^,^^60^^,^^67^^,^^77^ analyzed the effectiveness of psychosocial interventions on the quality of life of parents of cancer patients. Among these, 1 study on a family-centered intervention and 3 studies on parent-centered interventions showed that following psychosocial intervention, parents’ quality of life improved in areas including physical health, social/family well-being, emotional well-being, functional health, and spiritual health (*P* < 0.05).

## Discussion

This scoping review highlights the predominance of parenting-related psychosocial interventions available for cancer patients. These interventions are primarily focused on enhancing parenting skills, improving relationships within the family, facilitating communication, providing social support, improving family functioning, reducing distress, fostering adaptive coping strategies, and increasing children's understanding of their parents' cancer.

In terms of participants, nearly half of the interventions included parent-child dyads. For instance, the Enhancing Connections-Group intervention^81^ targeted communication and parenting issues faced by breast cancer patients, aiming to reduce maternal depression and anxiety, improve parenting behaviors (including quality, skills, and self-efficacy), and support children's behavioral and emotional adjustment to their mothers' illness. While these interventions effectively promote parent-child communication and alleviate emotional stress for both parents and children, a critical gap remains. Specifically, the psychological distress of cancer patients raising younger children who lack advanced communication skills has not been adequately addressed. This highlights an area for further development, as intervention programs must consider the unique needs of families with very young children or those with developmental delays.

Some studies implemented family-centered interventions, involving not only parents and children but also extended family members and friends.^66^^,^^73^ Partners often play a central role as caregivers, contributing significantly to parenting-related support while being at risk of their own psychological distress. These findings emphasize the need to consider thepsychosocial well-being of partners within the framework of parenting interventions, as their mental health directly impacts the family dynamic and caregiving effectiveness.

The geographic scope of the studies reveals a predominance of interventions conducted in Western countries such as the United States and Australia. Only one study^56^ explored culturally adapted interventions, demonstrating the unique challenges and benefits of tailoring psychosocial support to specific cultural contexts. Research suggests that cultural differences significantly influence cancer patients’ perceptions of their illness and their communication and needs within the family.^88^ For example, in Chinese culture, family harmony, mutual respect, and emotional care are highly valued. Negative emotions are often concealed to avoid causing distress to others.^89^ This tendency to suppress emotional expression underscores the need for culturally sensitive interventions that align with familial and societal norms. Current interventions often lack such specificity, underscoring the importance of developing personalized, culturally tailored parenting programs.

In terms of delivery methods, most interventions were delivered face-to-face, a mode that presents challenges for cancer patients due to the prolonged nature of treatment and the difficulty of attending in-person sessions during home-based recovery. Web-based interventions offer a promising alternative, effectively addressing parenting concerns, family functioning, and parenting self-efficacy while enhancing patient satisfaction and accessibility.^11^^,^^39^ Web-based programs are not constrained by time or location and require fewer resources, making them a practical solution for patients who need flexibility in scheduling and content delivery. Future research should focus on expanding and refining web-based interventions to allow cancer patients to access tailored support that aligns with their specific needs and circumstances.

In conclusion, while existing interventions demonstrate significant potential in addressing the parenting concerns of cancer patients, gaps remain in addressing the needs of younger children, cultural adaptation, and flexible delivery modes. Personalized, culturally specific, and web-based intervention models represent key areas for future development to improve accessibility, relevance, and outcomes for diverse patient populations. Furthermore, current research pays insufficient attention to cancer stage, and heterogeneity in disease stages may lead to variations in intervention effects among patients at different stages, affecting the generalizability of the conclusions. Therefore, future efforts should prioritize the design of personalized interventions tailored to specific disease stages, particularly for advanced-stage patients.

## Strengths and limitations

The main strengths of this study are reflected in the following aspects. First, through systematically reviewing and integrating various psychosocial interventions for parents with cancer, this research strictly adheres to the objectives of a scoping review, establishing a structured knowledge framework and presenting the findings clearly through tables and other formats. This scoping review reveals that existing psychosocial interventions rarely systematically incorporate culturally adaptive designs, particularly in Asian countries, where interventions tailored to local cultural contexts remain notably scarce. This finding highlights a significant gap in current research and practice, while also providing a clear direction and urgent rationale for developing future interventions that integrate cultural sensitivity and align with region-specific psychosocial characteristics. Second, this scoping review found that existing interventions primarily target children aged 6 to 17, with a focus on enhancing parent-child communication. However, specialized intervention programs for families with younger children, particularly those who have not yet developed mature verbal communication skills, remain notably scarce. This gap underscores a critical direction for future research: there is an urgent need to develop tailored support strategies for parents with cancer with young children (e.g., aged 0–6) . Approaches such as non-verbal interaction, parent-child play therapy, or family ritual-building—adapted to the developmental stages of the children—should be designed to help these families maintain emotional bonds and parenting functions during such challenging periods. Finally, this study significantly expands the diversity of evidence sources by systematically integrating empirical research from the Chinese context. Such literature is often overlooked in international English-language reviews yet accurately reflects the unique psychosocial and cultural background of China as a country with a high cancer burden, holding significant regional representativeness and clinical reference value. Although future searches of non-English databases could further enrich the global evidence base, this study focuses on systematically integrating Chinese-language evidence precisely to address the current underrepresentation of this population in international evidence syntheses, providing a critical foundation for subsequent cross-cultural comparisons and the optimization of intervention models.

However, the following limitations should be considered when interpreting the findings of this scoping review. First, to define the search boundaries, this study focused on literature published in Chinese and English. While this may limit the global coverage of evidence, it ensures the depth of retrieval and the relevance to Chinese-speaking populations with a high disease burden. Second, as a scoping review, its primary aim is to systematically map the overall landscape of the field rather than to grade study quality or quantitatively synthesize effects. Consequently, the evidence presented exhibits natural heterogeneity in study design, intervention approaches, and outcome measures. Additionally, some original studies inadequately reported key demographic information such as cancer stage, which constrained in-depth analysis based on disease stage. Future research should systematically collect staging data to refine the analysis of intervention effects and may build on this review to conduct systematic reviews and meta-analyses, thereby further quantifying the effects of key interventions.

## Conclusions

This scoping review evaluated the varied use of psychosocial interventions designed to assist parents with cancer in parenting underage children and identified several gaps in the literature. The included studies originated from seven countries, with 90.6% of the interventions developed for Western populations, highlighting a significant lack of research focused on parents with cancer in Asian countries. While the interventions demonstrated positive effects on parents with cancer, cultural differences in communication and family needs underscore the necessity of developing targeted and culturally adaptive psychosocial interventions for this vulnerable population.

Furthermore, the review emphasizes the potential of web-based intervention programs. These interventions are not limited by time or location, making them highly accessible and flexible for patients with cancer who may face logistical challenges in attending face-to-face sessions. Future research should prioritize culturally tailored and web-based psychosocial interventions to address the diverse needs of cancer-affected families and improve outcomes across different cultural and geographic contexts.

## CRediT authorship contribution statement

**Yingchun Li:** Conceptualization, Methodology, Formal analysis, Writing - Original Draft, Writing - Review & Editing. **Meichan Chong:** Conceptualization, Writing - Review & Editing, Supervision. **Pinglei Chui:** Conceptualization, Writing - Review & Editing, Supervision. **Lin Mo:** Methodology, Supervision. **Liande Tao:** Conceptualization, Formal analysis. **Yuman Yuan:** Methodology, Formal analysis. **Rong Liao:** Methodology, Formal analysis. **Haosong Ling:** Methodology, Formal analysis. **Qiaoli Zhong:** Methodology, Formal analysis. All authors have read and approved the final manuscript.

## Ethics statement

Not required.

## Data availability statement

The data supporting the findings of this study are available within the article and supplementary material.

## Declaration of generative AI and AI-assisted technologies in the writing process

No AI tools/services were used during the preparation of this work.

## Funding

This study received no external funding.

## Declaration of competing interest

The authors declare no conflict of interest.
