# Supplementary material for: Psychosocial interventions to support parenting among parents with cancer: A scoping review
Source: Asia Pac J Oncol Nurs. 2026 Feb 19;13:100921. doi: 10.1016/j.apjon.2026.100921 (PMC13022640; doi:10.1016/j.apjon.2026.100921)
Supplement: Multimedia component 1 [file mmc1.docx]

**Search Strategy**

Medline

Searched June 30, 2025, Retrieved 2703 Results. “neoplasm / exp” OR (neoplasm OR cancer OR tumor OR oncol*). ti, ab AND (paternal OR coparent* OR co-parent* OR parent* OR caregiver OR father* OR mother). ab, ti. AND (child* OR adolescent* OR teen* OR young OR infan* OR newborn* OR toddler* OR preschoolers OR youngsters OR offspring OR youth* OR minor child* OR minor* OR underage child* OR underage). ab, ti. AND (psycho* OR social OR practical OR emotional OR information). ab, ti. AND (evaluation OR intervention). ab. ti.

Web of Science

Searched June 30, 2025, Retrieved 896 Results. (neoplasm OR cancer OR tumor OR oncol*). ti, ab AND (paternal OR coparent* OR co-parent* OR parent* OR caregiver OR father* OR mother). ab, ti. AND (child* OR adolescent* OR teen* OR young OR infan* OR newborn* OR toddler* OR preschoolers OR youngsters OR offspring OR youth* OR minor child* OR minor* OR underage child* OR underage). ab, ti. AND (psycho* OR social OR practical OR emotional OR information). ab, ti. AND (evaluation OR intervention). ab. ti.

EMBASE

Searched June 30, 2025, Retrieved 1013 Results. ( “neoplasm”:ti, ab, kw OR “cancer”: ti, ab, kw OR oncol*:ti, ab, kw) AND (paternal: ti, ab, kw OR coparent*: ti, ab, kw OR co-parent*: ti, ab, kw OR parent*: ti, ab, kw OR caregiver : ti, ab, kw OR father* : ti, ab, kw OR mother: ti, ab, kw) OR (child*: ti, ab, kw OR adolescent*: ti, ab, kw OR teen*: ti, ab, kw OR young: ti, ab, kw OR infan*: ti, ab, kw OR newborn*: ti, ab, kw OR preschoolers: ti, ab, kw OR youngsters : ti, ab, kw OR offspring: ti, ab, kw OR youth*: ti, ab, kw OR minor child*: ti, ab, kw OR minor* : ti, ab, kw OR underage child* : ti, ab, kw OR underage: ti, ab, kw) AND (psycho*: ti, ab, kw OR social: ti, ab, kw) AND (evaluation : ti, ab, kw OR intervention: ti, ab, kw).

CINAHL

Searched June 30, 2025, Retrieved 886 Results. ((MH “neoplasm”)) OR TI (neoplasm OR cancer OR tumor OR oncol*) OR AB (neoplasm OR cancer OR tumor OR oncol*) AND MH (fathers) OR TI (paternal or or coparent* or co-parent* or parent* or caregiver or father* or mother) OR AB (paternal or or coparent* or co-parent* or parent* or caregiver or father* or mother) OR TI (child* or adolescent* or teen* or young or infan* or newborn* or preschoolers or youngsters or offspring or youth* or minor child* or minor* or underage child* or underage) OR AB (child* or adolescent*or teen* or young or infan* or newborn* or preschoolers or youngsters or offspring or youth* or minor child* or minor* or underage child* or underage) AND (TI (psycho* or social) OR AB (psycho* or social) )AND (TI (evaluation or intervention) OR AB (evaluation or intervention))

PsycINFO

Searched June 30, 2025, Retrieved 1254 Results. (TI (neoplasm OR cancer OR tumor OR oncol*) OR AB (neoplasm OR cancer OR tumor OR oncol*) OR MA tumor OR MA neoplasm) AND (AB (paternal or or coparent* or co-parent* or parent* or caregiver or father* or mother) OR TI (paternal or coparent* or co-parent* or parent* or caregiver or father* or mother) OR MA fathers) OR AB (child* or adolescent*or teen* or young or infan* or newborn*or preschoolers or youngsters or offspring or youth* or minor child* or minor* or underage child* or underage) OR TI (child* or adolescent*or teen* or young or infan* or newborn*or preschoolers or youngsters or offspring or youth* or minor child* or minor* or underage child* or underage) AND (AB (psycho* or social) OR TI (psycho* or social))AND (AB (evaluation or intervention) OR TI (evaluation or intervention))

Social Work Abstracts

Searched June 30, 2025, Retrieved 86 Results. (neoplasm OR cancer OR tumor OR oncol*). ti, ab AND (paternal OR coparent* OR co-parent* OR parent* OR caregiver OR father* OR mother). ab, ti. AND (child* OR adolescent* OR teen* OR young OR infan* OR newborn* OR toddler* OR preschoolers OR youngsters OR offspring OR youth* OR minor child* OR minor* OR underage child* OR underage). ab, ti. AND (psycho* OR social OR practical OR emotional OR information). ab, ti. AND (evaluation OR intervention). ab. ti.

CENTRAL

Searched June 30, 2025, Retrieved 978 Results. ( “neoplasm”:ti, ab, kw OR “cancer”: ti, ab, kw OR oncol*:ti, ab, kw) AND (paternal: ti, ab, kw OR coparent*: ti, ab, kw OR co-parent*: ti, ab, kw OR parent*: ti, ab, kw OR caregiver : ti, ab, kw OR father* : ti, ab, kw OR mother: ti, ab, kw) OR (child*: ti, ab, kw OR adolescent*: ti, ab, kw OR teen*: ti, ab, kw OR young: ti, ab, kw OR infan*: ti, ab, kw OR newborn*: ti, ab, kw OR preschoolers: ti, ab, kw OR youngsters : ti, ab, kw OR offspring: ti, ab, kw OR youth*: ti, ab, kw OR minor child*: ti, ab, kw OR minor* : ti, ab, kw OR underage child* : ti, ab, kw OR underage: ti, ab, kw) AND (psycho*: ti, ab, kw OR social: ti, ab, kw) AND (evaluation : ti, ab, kw OR intervention: ti, ab, kw).

CNKI

Searched June 30, 2025, Retrieved 345 Results. (SU %= '癌' OR SU %= '肿瘤') AND (SU %= '父母' OR SU %= '父亲' OR SU %= '母亲' OR SU %= '双亲' OR SU %= '爸爸' OR SU %= '妈妈') AND (SU %= '子女' OR SU %= '孩子' OR SU %= '青少年' OR SU %= '儿童' OR SU %= '未成年子女' OR SU %= '幼儿' OR SU %= '婴儿') AND SU %= '干预'

Wan fang database

Searched June 30, 2025, Retrieved 393 Results. (主题:(癌) or 主题:(肿瘤) ) and (主题:(未成年孩子) or 主题:(未成年子女) or 主题:(孩子)) and (主题:(母亲) or 主题:(父亲) or 主题:(父母) or 主题:(双亲))
